# A Preliminary Single-Cell RNA-Seq Analysis of Embryonic Cells That Express *Brachyury* in the Amphioxus, *Branchiostoma japonicum*

**DOI:** 10.3389/fcell.2021.696875

**Published:** 2021-07-15

**Authors:** Noriyuki Satoh, Hitoshi Tominaga, Masato Kiyomoto, Kanako Hisata, Jun Inoue, Koki Nishitsuji

**Affiliations:** ^1^Marine Genomics Unit, Okinawa Institute of Science and Technology Graduate University, Okinawa, Japan; ^2^Tateyama Marine Laboratory, Marine and Coastal Research Center, Ochanomizu University, Chiba, Japan; ^3^Atmosphere and Ocean Research Institute, University of Tokyo, Chiba, Japan

**Keywords:** scRNA-seq analysis, amphioxus embryos, two *Brachyury* genes, notochord, somites, blastopore, tail bud, *Bra2* orthology

## Abstract

Among chordate taxa, the cephalochordates diverged earlier than urochordates and vertebrates; thus, they retain unique, primitive developmental features. In particular, the amphioxus notochord has muscle-like properties, a feature not seen in urochordates or vertebrates. Amphioxus contains two *Brachyury* genes, *Bra1* and *Bra2*. *Bra2* is reportedly expressed in the blastopore, notochord, somites, and tail bud, in contrast to a low level of *Bra1* expression only in notochord. To distinguish the expression profiles of the two *Brachyury* genes at the single-cell level, we carried out single-cell RNA-seq (scRNA-seq) analysis using the amphioxus, *Branchiostoma japonicum*. This scRNA-seq analysis classified *B. japonicum* embryonic cells into 15 clusters at developmental stages from midgastrula to early swimming larva. *Brachyury* was expressed in cells of clusters 4, 5, 8, and 9. We first confirmed that cluster 8 comprises cells that form somites since this cluster specifically expresses four myogenic factor genes. Cluster 9 contains a larger number of cells with high levels of *Bra2* expression and a smaller number of cells with *Bra1* expression. Simultaneous expression in cluster 9 of tool-kit genes, including *FoxA*, *Goosecoid*, and *hedgehog*, showed that this cluster comprises cells that form the notochord. Expression of *Bra2*, but not *Bra1*, in cells of clusters 4 and 5 at the gastrula stage together with expression of *Wnt1* and *Caudal* indicates that clusters 4 and 5 comprise cells of the blastopore, which contiguously form the tail bud. In addition, *Hox1, Hox3*, and *Hox4* were highly expressed in *Bra2*-expressing clusters 4, 5, 8, and 9 in a temporally coordinated manner, suggesting roles of anterior Hox genes in specification of mesodermal organs, including somites, notochord, and tail bud. This scRNA-seq analysis therefore highlights differences between the two *Brachyury* genes in relation to embryonic regions in which they are expressed and their levels of expression. *Bra2* is the ancestral *Brachyury* in amphioxus, since expression in the blastopore is shared with other deuterostomes. On the other hand, *Bra1* is a duplicate copy and likely evolved a supplementary function in notochord and somite formation in the *Branchiostoma* lineage.

## Introduction

The origin and evolution of chordates are two of the most intriguing evo-devo research subjects of metazoans (Holland et al., 2015; Lowe et al., 2015; Satoh, 2016; Gee, 2018). Among the three chordate taxa, cephalochordates, urochordates (tunicates), and vertebrates, cephalochordates were the first group to diverge (Bourlat et al., 2006; Delsuc et al., 2006; Putnam et al., 2008); thus, they exhibit unique features during embryogenesis, while sharing others with echinoderms and hemichordates, and still others with urochordates and vertebrates (Whittaker, 1997: Yu et al., 2007; Bertrand and Escriva, 2011; Holland et al., 2015).

**FIGURE 1:**
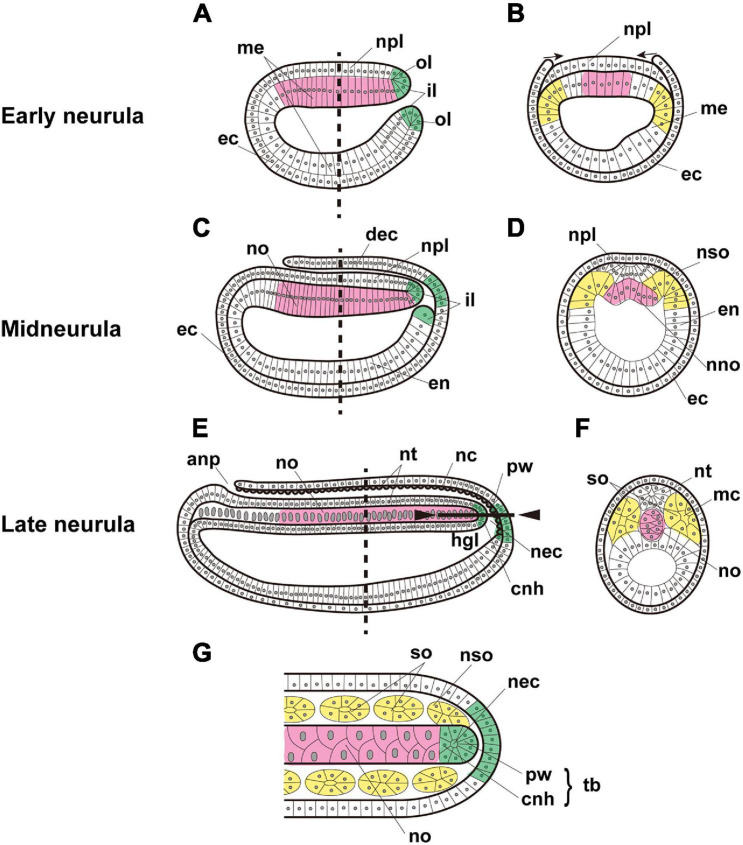
Expected expression of *Brachyury* genes in the notochord (magenta), somites (yellow), and tail bud (green) of amphioxus embryos. Early neurula, **(A)** sagittal section, and **(B)** cross section at the location shown by the dashed line in panel **(A)**. Arrows indicate the migration of ectoderm across the neural plate. Midneurula, **(C)** sagittal section, and **(D)** cross section at the level shown by the dashed line in panel **(C)**. Cross section showing somites and notochord beginning to form. Late neurula, **(E)** sagittal section, **(F)** cross section at the level shown by the dashed line in panel **(E)**, and **(G)** frontal section of the region indicated by the solid line between arrowheads in panel **(E)**. In alphabetical order, abbreviations are as follows: anp, anterior neuropore; cnh, chordoneural hinge; dec, dorsal ectoderm; ec, ectoderm; en, endoderm; hgl, hindgut lumen; il, inner lip of blastopore; mc, myocoel; me, mesendoderm; nc, neurocoel; nec, neurenteric canal; nno, nascent notochord; no, notochord; npl, neural plate; nso, nascent somite; nt, neural tube; ol, outer lip of blastopore; pw, posterior wall of neurenteric canal; so, somite; tb, tail bud. The original drawing of embryos by Schubert et al. (2001) is cited.

In particular, the developmental mode and properties of the cephalochordate notochord are unique, compared with those of tunicates and vertebrates (Satoh et al., 2012). First, the cephalochordate notochord is formed at roughly the neurulation stage by “pouching-off” from the dorsal region of the archenteron, like somites, which are also pinched off from both the left and right sides of the archenteron (Conklin, 1932; Hirakow and Kajita, 1994; Figure 1). This contrasts with urochordate and vertebrate notochords, which are formed by convergent extensions of precursor cells that are bilaterally positioned in the early embryo (Munro and Odell, 2002). Second, the cephalochordate notochord has muscle-like properties and possesses myofibrils (Ruppert, 1997; Suzuki and Satoh, 2000; Urano et al., 2003). This also contrasts with urochordate and vertebrate notochords, which do not possess any muscle-like properties, except for the posterior part of the *Ciona* embryonic notochord (Cao et al., 2019). Vacuolation within notochord cells provides both stiffness and an increase in cell volume in ascidians and vertebrates (Jiang and Smith, 2007).

We are interested in a T-box family transcription factor gene, *Brachyury*, and its role in formation of the notochord during chordate evolution (Satoh et al., 2012; Satoh, 2016). In non-chordate deuterostomes, *Brachyury* is expressed in the blastopore and stomodeum of early embryos and functions in morphogenetic movement of archenteron invagination and mouth invagination (Tagawa et al., 1998; Gross and McClay, 2001). In chordates, *Brachyury* is expressed in the notochord, and functions in development of this chordate-specific organ. We consider the former primary and the latter secondary expression and function of *Brachyury* (Satoh et al., 2012). Thus, the acquisition of secondary expression and function of *Brachyury* is a foundational research subject to decipher the origin and evolution of chordates.

As mentioned above, the cephalochordate notochord is unique from an evo-devo perspective. In addition, in contrast to the single copy present in genomes of deuterostomes, *Brachyury* was tandemly duplicated into *Bra1* and *Bra2* in cephalochordate genomes (Holland et al., 1995; Inoue et al., 2017). These genes are expressed in the blastopore of gastrula, notochord, somites, and the tail bud of neurulae (Holland et al., 1995; Terazawa and Satoh, 1997; Figure 1). However, due to high levels of sequence similarity, previous whole-mount *in situ* hybridization (WMISH) analyses failed to distinguish the expression profiles of *Bra1* and *Bra2.* Recently, Yuan et al. (2020) tackled this question using WMISH with probes from the divergent untranslated regions specific to each of the genes. They showed that the zygotic expression level of *Bra2* is much higher than that of *Bra1*, and that *Bra2* is highly expressed in the blastopore, tail bud, and notochord, while *Bra1* is weakly transcribed only in the notochord. In addition, a heterogenic transplantation assay of *cis*-regulatory sequences of *Bra1* and *Bra2* into the *Ciona* embryonic system demonstrated that the 5’ upstream sequence of *Bra2* contains higher enhancer activity in both the notochord and somites, compared to that of the 5’ upstream sequence of *Bra1* in the notochord (Tominaga et al., 2018). These results suggest that both *Bra1* and *Bra2* are *Brachyury* orthologs and that *Bra1* has diverged more. Nevertheless, many questions remain to be answered in order to understand evo-devo features of cephalochordate *Brachyury* and the notochord.

The recently developed technology of single-cell RNA sequencing (scRNA-seq) constitutes a powerful tool to categorize genes expressed in constituent cells of embryos, tissues, or organs on a cell-by-cell basis (Cao et al., 2019; Sharman et al., 1999; Stuart and Satija, 2019; Foster et al., 2020). We employed scRNA-seq analyses of amphioxus embryos to clarify how expression levels of *Bra1* and *Bra2* differ in embryonic regions.

## Results

### Clustering of Embryonic Cells

**FIGURE 2:**
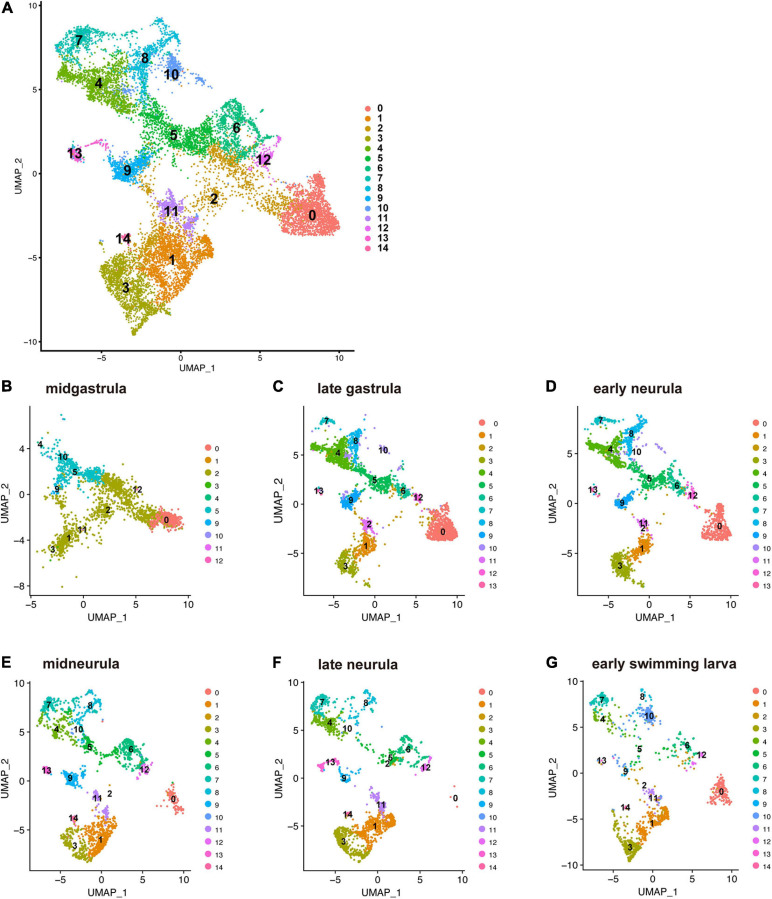
UMAP plots of early developmental stages of *Branchiostoma japonicum.* **(A)** UMAP plots based on a combined dataset of all six stages. **(B)** Midgastrula, **(C)** late gastrula, **(D)** early neurula, **(E)** midneurula, **(F)** late neurula, and **(G)** early swimming larva after alignment. A total of 14,016 single cells are colored by cluster identity. Fifteen populations (clusters 0–14) are detected across the six time points. As discussed in the text, cluster 0 corresponds to ectoderm (red brown area) since a marker gene that encodes type 1 keratin is expressed in the cluster. Clusters 1, 2, 3, 11, and 12 correspond to endoderm (light green and brown area) since *FoxA* and *Goosecoid*, markers for endoderm, are expressed in these clusters. Clusters 4, 5, 8, and 9 correspond to mesoderm (green and blue area), since *Brachyury* is expressed in these clusters. Clusters 6, 7, and 10 may be mesoderm, while clusters 13 and 14 remain to be characterized.

*Branchiostoma japonicum* embryos were cultured and collected for scRNA-seq analysis using the 10 × Genomics platform. We selected six embryonic stages from midgastrula to early swimming larva (Supplementary Table 1), on the basis of dynamic features of cell fate changes anticipated by previous studies (Bertrand and Escriva, 2011; Marletaz et al., 2018). Datasets from all six stages were integrated and clustered to identify cells with similar or different gene expression profiles across early development using Seurat (Butler et al., 2018; Stuart et al., 2019). In total, transcriptomes of 14,016 cells were included in this analysis. Upon integration of the datasets and visualization with UMAP (Uniform Manifold Approximation and Projection) dimensionality reduction, we identified 15 major cell clusters, numbered 0 to 14, based on marker gene expression (Figure 2A and Supplementary Table 2). Identification of 15 clusters resulted from the resolution parameter, 0.5, a conservative setting, used in the clustering step of the analysis.

The present characterization of 15 embryonic-cell clusters encountered two problems. The first was the presence of fewer clusters in the midgastrula stage than in the late gastrula and later stages (Figure 2B). This might occur because midgastrula consists of only three germ layers, outer ectoderm, inner endoderm, and intermediate mesoderm, and specification/differentiation of embryonic cells may not have occurred for sufficient clustering of cells (Figure 2B). Since the gene encoding type 1 keratin, a marker gene for epidermis specification (Petillon et al., 2017), is specifically expressed in cluster 0, this cluster corresponds to ectoderm (red brown area in Figure 2B). As described below, clusters 1, 2, 3, 11, and 12 express *FoxA* and *Goosecoid*, markers for endoderm, so these clusters (Figure 2B) constitute endoderm. *Brachyury* is expressed in clusters 4, 5, 8, and 9 (see below), indicating that they are mesoderm territory (Figure 2B). The number of clusters therefore increased as development proceeded (compare Figure 2B to Figures 2C,D). The second was the scarceness of cells that constitute each cluster at early swimming larva (Figure 2G). It may be that the available scRNA-seq reads were inadequate to cover a large number of constituent cells of larvae at the stage. However, data from the remaining four stages (late gastrula, early neurula, midneurula, and late neurula) contained enough RNA for further characterization of cell clusters that comprise early amphioxus embryos.

### A Cluster of Cells With Myogenic Factor Gene Expression: Cluster 8

Spatial expression profiles of developmentally relevant genes, especially for transcription factors and signaling molecules, have been examined extensively in amphioxus embryos by cDNA cloning and WMISH analysis (Holland et al., 1995; Schubert et al., 2001). Expression profiles of most tool-kit genes did not appear specific to certain embryonic regions, but spanned multiple regions, probably due to a low level of specification in amphioxus early embryonic cells. One example of specific spatial expression was seen among myogenic factor (MF) genes, which are expressed exclusively in the developing paraxial mesoderm or somites (Schubert et al., 2003; Aase-Remedios et al., 2020). Since somites are embryonic organs with expected *Brachyury* expression, and since MF genes are good candidates to examine the appropriateness of our clustering method of amphioxus embryonic cells, we first explored cells of clusters that expressed MF genes.

**FIGURE 3:**
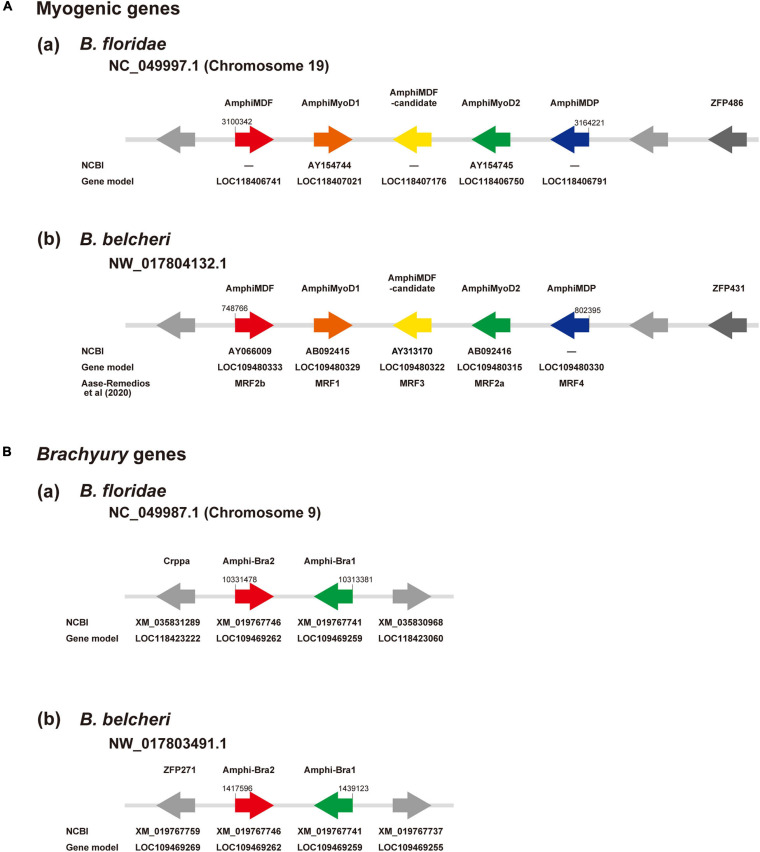
**(A)** Myogenic factor genes and **(B)** *Brachyury* genes in amphioxus genomes. **(A)** Genomic region containing a cluster of myogenic factor genes in (a) *Branchiostoma floridae* and (b) *B. belcheri*. In scaffold NC_049997.1 of chromosome 19 in *B. floridae* and NW_017804132.1 in *B. belcheri*, there is a cluster that contains five genes for myogenic factors, from left to right, MDF (red), MyoD1 (brown), a candidate myogenic factor gene (yellow), MyoD2 (green), and myoblast determination protein (blue). Two undefined genes are shown in gray and a zinc finger protein gene is shown in dark gray. Arrows indicate the direction of transcription. **(B)** Genomic region containing two *Brachyury* genes, *Bra2* (red) and *Bra1* (green), in (a) *Branchiostoma floridae* scaffold NC_049987.1 of chromosome 9 and (b) *B. belcheri* scaffold NW_017803491. Arrows indicate the direction of transcription.

The amphioxus MF gene was duplicated in a specific manner in this lineage, independently of the vertebrate MF gene duplication into four copies, *MyoD, myogenin*, *Myf5*, and *MRF4* (Araki et al., 1996; Schubert et al., 2003; Tan et al., 2014). Recently, Aase-Remedios et al. (2020) reported five MF genes in *Branchiostoma* genomes. Here, we independently examined the number of MF genes in amphioxus. Surveying a newly assembled genome of *B. floridae* (Simakov et al., 2020), we found a cluster of five MF-related genes in scaffold NC_049997.1 of chromosome 19 (Figure 3Aa). They are *MDF* (gene model ID, LOC118406741), *MyoD1* (AY154744; LOC118407021), an uncharacterized copy (LOC118407176, tentatively called *MDF-candidate*), *MyoD2* (AY154745; LOC118406750), and a gene for myoblast determination protein (LOC118406791, tentatively called *MDP*) (Figure 3Aa). A survey of the *Branchiostoma belcheri* genome (Huang et al., 2014) showed an orthologous cluster with five genes in scaffold NW_017804132.1 (Figure 3Ab), *MDF* (AY066009; LOC109480333), *MyoD1* (AB092415; LOC109480329), *MDF-candidate* (AY313170; LOC109480322), *MyoD2* (AB092416; LOC109480315), and *MDP* (LOC109480330) (Figure 3Ab). Molecular phylogeny using ORTHOSCOPE (Inoue and Satoh, 2018) showed that (a) *MDF* and *MyoD2* form a clade that is sister to *MyoD1*, and (b) *MDF-candidate* is an early branch of amphioxus MF genes and *MDP* (Supplementary Figure 1A). These results coincided well with those of Aase-Remedios et al. (2020); *MDF, MyoD1, MDF-candidate, MyoD2*, and *MDP* correspond to *MRF2b, MRF1, MRF3, MRF2a*, and *MRF4* of Aase-Remedios et al. (2020), respectively (Figure 3Ab).

**FIGURE 4:**
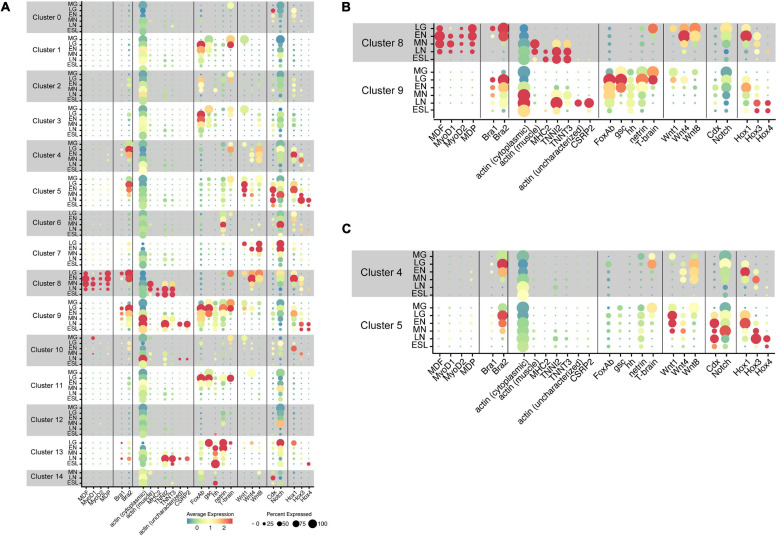
Transcriptome trajectories for 15 clusters comprising early stages of *Branchiostoma japonicum* embryos. Dot size represents the percentage of cells that express genes for transcription factors, signaling molecules, and structural proteins. Dot color shows the averaged level of expression. MG, midgastrula; LG, late gastrula; EN, early neurula; MN, midneurula; LN, late neurula; ESL, early swimming larva. **(A)** A summary figure. **(B)** Enlargement of clusters 8 and 9. **(C)** Enlargement of clusters 4 and 5.

With the five amphioxus MF genes as probes, we carried out scRNA-seq analysis and results obtained are shown in UMPA plots (Supplementary Figure 2) and dot plots (Figure 4). We found that (a) of the five MF genes, a cell cluster expressing *MDF* (Supplementary Figure 2A), *MyoD1* (Supplementary Figure 2B), *MyoD2* (Supplementary Figure 2C), and *MDP* (Supplementary Figure 2D) was evident, but no clusters expressed *MDF-candidate* at a detectable level (data not shown); (b) all four MF genes were expressed in cells that comprise cluster 8 (Figure 4A and Supplementary Figure 2); (c) the expression level of *MDF* and *MDP* was higher than those of *MyoD1* and *MyoD2* (Figures 4A,B); (d) expression of *MDF* and *MDP* became evident at the late gastrula stage and continued until the late neurula stage (Figures 4A,B); (e) expression of *MyoD1* was detected at the early, mid, and late neurula stage (Figures 4A,B); and (f) expression of *MyoD2* was detected at the four earlier stages (Figures 4A,B). These results indicate that cells of cluster 8 comprise the developing somites, consistent with results of previous studies showing that amphioxus MF genes are expressed exclusively in embryonic cells that give rise to paraxial muscle or somites at gastrula and neurula stages (Schubert et al., 2003; Urano et al., 2003; Aase-Remedios et al., 2020). Based on their higher expression levels, *MDF* and *MDP* likely function in myogenesis of amphioxus embryos rather than *MyoD1* and *MyoD2*, although this should be further examined experimentally since Aase-Remedios et al. (2020) reported expression of all MF genes in *Branchiostoma* embryos. Nevertheless, the appropriateness of our clustering method was also supported by these results.

### Cluster of Cells With *Bra1* and *Bra2* Expression: Cluster 9

As in the case of MF genes, we first examined localization of *Bra1* and *Bra2* in amphioxus genomes and their molecular phylogenetic relationships among deuterostome T-box family members for basic information following scRNA-seq analysis. *Bra1* and *Bra2* were present in scaffold NC_049987.1 of chromosome 9 in the *B. floridae* genome (Figure 3Ba) and in scaffold NW_017803491.1 in the *B. belcheri* genome (Figure 3Bb). They exhibit a head-to-head orientation, and there were no other T-box family members in this genomic region. Molecular phylogeny using ORTHOSCOPE demonstrated the presence of seven T-box subfamily members in lancelet genomes, TBX20, TBX15/18/22/, TBX1/10, TBX4/5, TBX2/3, TBR1/TBX21, and Bra (Supplementary Figure 1B). Duplication into *AmphiBra1* and *AmphiBra2* appears to be an amphioxus-specific event (Inoue et al., 2017). A sister clade to *Bra* clade is *Tbrain/Tbx21*. *Tbrain1* is apparently expressed in the dorsal region of invaginating archenteron in gastrulae, and then in the preoral-pit region of larvae (Satoh et al., 2002), suggesting overlapping expression that of *Bra* and *Tbrain1* in gastrulae.

The present scRNA-seq analysis showed that *Bra* is expressed in cells of clusters 4, 5, 8, and 9 (Figure 4 and Supplementary Figure 3). A small number of cells expressed *Bra* weakly in cluster 13 (Figure 4), and the property of this cluster should be characterized in future studies. Expression of *Bra2* (Supplementary Figure 3B) was much higher than that of *Bra1* (Supplementary Figure 3A) both at the level of transcription and in embryonic regions in which the gene is expressed (Figure 4). The occurrence of cells with *Bra1* expression was detected in clusters 8 and 9 at late gastrula and in cluster 9 at early neurula and midneurula and became undetectable by the late neurula stage (Figure 4B). In addition, cluster 9 contained a larger number of cells with high *Bra2* expression and a smaller number of cells with *Bra1* expression (Figure 4B).

As shown in the previous section, cluster 8 pertains to the developing somites. This indicates that cluster 9 comprises cells of the developing notochord. Therefore, at the late gastrula stage, *Bra1* is expressed in both the presumptive notochord and the presumptive paraxial muscle. These results basically coincide with those of Yuan et al. (2020), although they reported no detectable level of *Bra1* expression at the gastrula stage and in somites (cluster 8) (Yuan et al., 2020).

### Clusters 8 and 9 Express Muscle Structural Genes

As already mentioned, the cephalochordate notochord has muscle-like properties. Expression of muscle structural genes, including actin and myosin heavy chain, has been reported not only in somites, but also in the notochord (Suzuki and Satoh, 2000; Urano et al., 2003; Inoue and Satoh, 2018). We first examined whether cells of cluster 8 (somites) express muscle structural genes to confirm that this cluster comprises cells for the developing somites. Three genes examined for this purpose were cytoplasmic actin (LOC109483419) (Supplementary Figure 4A), paraxial muscle actin (LOC109481400) (Supplementary Figure 4B), and myosin heavy chain (MHC2) (LOC109470521) (Supplementary Figure 4C). As expected, considerable cytoplasmic actin gene expression was detected in most cell clusters through the six stages (Figure 4). In contrast, the gene for paraxial muscle actin was detected in cluster 8 at midneurula and later stages (Figures 4A,B), and *MHC*2 gene was detected in cluster 8 at late neurula and early larva stages (Figures 4A,B). Therefore, the affiliation of cluster 8 with somites was confirmed by expression of paraxial muscle structural genes. Earlier expression of *MDF* and *MDP* than paraxial muscle structural genes (Figure 4B) suggests regulatory control of MF genes over transcription of structural genes.

The cephalochordate notochord has been reported to express muscle structural genes, some shared with somites while some others are specific to the notochord (Suzuki and Satoh, 2000; Urano et al., 2003; Inoue and Satoh, 2018). Although detailed identification and characterization of muscle structural genes in the developing cephalochordate notochord using scRNA-seq data are the subject of future research, here we examined four genes, gene for troponin I2 (TNNI2; LOC109469528) (Supplementary Figure 4D), for troponin T3 (TNNT3; LOC109481859) (Supplementary Figure 4E), for possible notochord-specific actin (not characterized yet) (LOC109482101) (Supplementary Figure 4F), and for cysteine- and glycine-rich protein 2 (CSRP2) (LOC109481702) (Supplementary Figure 4G). *CSRP2* encodes a group of LIM domain proteins, a cephalochordate homolog of which was reported to express in the developing notochord (Urano et al., 2003). High levels of *troponin I2* and *troponin T3* expression were detected in cluster 8 at late neurula and early swimming larva stages (Figure 4B). In addition, high *troponin I2* expression was evident in cluster 9 at late neurula stages (Figure 4B). On the other hand, expression of the uncharacterized actin gene and *CSRP2* was detected only in cells of cluster 9 at late neurula (Figure 4B). Therefore, this analysis suggests the unique properties of the cephalochordate notochord, in which genes for several muscle structural proteins are likely controlled by *Brachyury*, but not by *MDF/MyoD*, since *MDF/MyoD* is not expressed in cells of cluster 9.

### Characterization of Notochord Cluster 9 With Other Transcription Factor Genes

Previous cDNA cloning studies of amphioxus genes followed by WMISH analysis demonstrated that various genes for transcription factors and signaling molecules are expressed in the embryonic region that gives rise to the notochord. We here examined scRNA-seq data of four genes, namely, *FoxAb* (LOC10948147) (Supplementary Figure 5A), *sonic hedgehog* (LOC10947428) (Supplementary Figure 5B), *goosecoid* (LOC109470978) (Supplementary Figure 5C), and *netrin* (LOC109462789) (Supplementary Figure 5D).

*FoxA* and *Brachyury* function in a coordinated manner to form endomesoderm in deuterostome embryos (e.g., Imai et al., 2006; Oliveri et al., 2006). *Branchiostoma floridae* contains two *FoxA* genes, *FoxAa* and *FoxAb* (Yu et al., 2008). Shimeld (1997) reported that transcripts of *FoxAb* are detected in the blastopore, endoderm, and notochord in amphioxus neurulae. The present scRNA-seq analysis showed that cells with *FoxAb* expression became detectable as early as the midgastrula stage in cluster 9 (Figure 4 and Supplementary Figure 5A). In late gastrula, the high level of expression was found in clusters 1, 2, 3, 9, and 11 (Figure 4), and this expression remained in early, mid-, and late neurula (Figure 4). This indicates that cells of cluster 9 express *Bra2* and *FoxAb* simultaneously. Clusters 1, 2, 3, and 11 occupy a closely associated region of UMAP plots (Figure 2), and because in addition to *FoxAb*, an anterior endodermal marker of amphioxus embryos, *goosecoid* (Onai et al., 2015), is expressed in these clusters (Figure 4, see below), cells of clusters 1, 2, 3, and 11 were thought to comprise endoderm and its derivatives.

*Goosecoid* (*gsc*) encodes a homeodomain-containing transcription factor that is expressed in the vertebrate head organizer and that can induce a secondary axis when expressed ectopically. Neidert et al. (2008) and Onai et al. (2015) reported that the expression of *Amphioxus gsc* is initially localized during gastrulation to the endomesoderm layer of the dorsal lip of the blastopore. Then, *gsc* is expressed in the anterior and dorsal endoderm and in the presumptive notochord that underlies the presumptive central nervous system (Onai et al., 2015). This scRNA-seq analysis showed that *gsc* is expressed in cells of cluster 9 at midgastrula, late gastrula, and early neurula (Figure 4B and Supplementary Figure 5C), further supporting the notion that cluster 9 consists of cells for the developing notochord. *gsc* expression was also detected in clusters 1, 3, and 11, suggesting that these clusters are derived from endoderm (Figure 4B and Supplementary Figure 5C).

A single amphioxus hedgehog gene, *hh*, is expressed in the notochord and ventral neural tube, embryonic tissues that express Sonic-type genes in vertebrates (Shimeld, 1999; Ono et al., 2018; Zhu et al., 2020). Our scRNA-seq analysis detected cells with *hh* expression in cluster 9 at late gastrula and later stages (Figure 4B and Supplementary Figure 5B), indicating that cluster 9 comprises cells that form the notochord. A high level of *hh* expression was detected in cluster 13 at early neurula and later stages (Figure 4 and Supplementary Figure 5B). This suggests that cluster 13 contains cells of neural tube.

An amphioxus *netrin* gene is expressed in midline structures of embryos, including the notochord and floor plate (Shimeld, 2000). This scRNA-seq analysis confirmed that *netrin* is expressed in cells of notochord cluster 9 (Figure 4B and Supplementary Figure 5D). In addition, *netrin* expression was detected in cells of clusters 6 and 13 at late gastrula and later stages (Figure 4 and Supplementary Figure 5D). As mentioned above, cluster 13 likely involves cells of the nervous system, and *netrin* expression in cluster 13 supports this notion. Cluster 6 likely pertains to mesoderm, but characterization of this cluster will be investigated in future studies.

*Tbrain1* is sister to *Bra* in the amphioxus T-box family (Supplementary Figure 1B), and this gene is reportedly expressed in the dorsal region of invaginating archenteron in gastrulae (Satoh et al., 2002). This scRNA-seq analysis showed that *Tbrain1* expression occurs in cells of cluster 8 at late gastrula and in cells of cluster 9 at mid- and late gastrula (Figure 4 and Supplementary Figure 5E). This indicates that cells of somite cluster 8 and notochord cluster 9 are derived from the dorsal region of the invaginating archenteron. This overlapping expression of *Tbrain1* with *Bra2* suggests some role of *Tbrain1* in the developing somite and notochord. A high level of *Tbrain1* expression was also detected in cells of clusters 1 and 11 at gastrula (Figure 4 and Supplementary Figure 5E), providing additional support for an association of clusters 1 and 11 with endoderm.

In summary, this scRNA-seq survey of five transcription factor genes almost coincides with results of previous WMISH studies and suggests that cluster 9, with *Brachyur*y expression, comprises cells of the developing notochord. Therefore, these transcription factor genes are likely involved either directly or indirectly in the formation of the notochord in amphioxus embryos.

### Co-expression of *Bra2* and *MDF*

The four MF genes, including *MDF*, are specifically expressed in cells of cluster 8 (somite) (Figure 4), and *Bra2* is also expressed in cells of clusters 8 (Figure 4), showing that presumptive somite cells simultaneously express MF genes and *Brachyury*. To examine co-expression of these genes at the single-cell level, we double-plotted cells with *MDF* expression and those with *Bra2* expression (Supplementary Figure 6). At late gastrula, co-expression was detected in most cells of cluster 8 (Supplementary Figure 6; LG). On the other hand, at early neurula, cells with only *MDF* expression appeared in the distal portion of the cluster (Supplementary Figure 6; EN). As embryos further developed to mid- and late neurula stage, the expression level of *Bra2* decreased (Figure 4B) and cluster 8 contained more cells with only *MDF* expression (Supplementary Figure 6; MN and LN). These results suggest that at the single-cell level, cells of cluster 8 utilize MF genes and *Brachyury* for specification as presumptive somite cells at the gastrula stage, while later at the neurula stage they use only MF genes for specification.

### Characterization of Clusters With *Bra2* Expression: Clusters 4 and 5

As mentioned above, embryonic regions with *Bra2* expression were comparatively broad because this gene is highly expressed not only in notochord cluster 9 and somite cluster 8, but also in clusters 4 and 5 (Figure 4 and Supplementary Figure 3). Cells of both clusters 4 and 5 began to express *Bra2* at the midgastrula stage and high level of expression was evident at late gastrula, although expression decreased as development proceeded, becoming undetectable at the late neurula stage (Figure 4C). Since *Brachyury* is reportedly first expressed in the blastopore region of gastrulae, and then in the tail bud, it is highly likely that clusters 4 and 5 correspond to these regions. Two clusters with *Bra2* expression suggest that the tail bud is composed of two regions with different properties. According to a review of amphioxus tool-kit genes involved in tail bud formation from the blastopore region (Holland, 2002), the tail bud is formed by combinatorial expression of *Brachyury*, several *Wnt* family members, *Caudal*, and *Notch*. Therefore, we examined whether cells of clusters 4 and 5 express *Wnt*, *Caudal*, and *Notch.*

A previous report identified eight members of the *Wnt* family in *B. floridae*: *Wnt1*, *Wnt3, Wnt4, Wnt5, Wnt6, Wnt7, Wnt8*, and *Wnt11* (Schubert et al., 2001). Among these, *Wnt8* was first expressed around the blastopore, followed by *Wnt1* (Schubert et al., 2001). Since an improved assembly of the *B. floridae* genome has been published (Simakov et al., 2020), we reexamined *Wnt* members in the *B. floridae* genome and found 12 *Wnt* family members: *Wnt1*, *Wnt2*, *Wnt3, Wnt4, Wnt5, Wnt6, Wnt7, Wnt8, Wnt9, Wnt10, Wnt11*, and *Wnt16* (Supplementary Figure 7). Our scRNA-seq analysis demonstrated distinct expression of *Wnt1*, *Wnt4*, and *Wnt8* in *B. japonicum* embryos (Supplementary Figure 8), while *Wnt3* and *Wnt7* were expressed at low levels (Supplementary Figure 8). A high level of *Wnt1* expression was found in cluster 5 at stages from late gastrula to midneurula (Figures 4A,C), and moderate *Wnt8* expression in cluster 4 at stages from late gastrula to midneurula (Figures 4A,C). *Wnt4* was expressed in clusters 4 and 5, but the expression level was lower than those of *Wnt1* and *Wnt8* (Figures 4A,C). These changes are consistent with previously reported expression patterns of *Wnt* genes (Holland et al., 2000; Schubert et al., 2000). Expression profiles of the three *Wnt* genes in the two clusters, therefore, are not identical. Cluster 5 contains cells with higher expression of *Wnt1* and cluster 4 has higher expression of *Wnt8* (Figures 4A,C), suggesting that the two clusters have different properties.

Amphioxus *Caudal* (*Cdx*) was reportedly expressed in the posterior mesendoderm and neural plate of early neurula and in the posterior endoderm, the walls of the neurenteric canal, and the posterior part of the nerve cord in late neurula (Brooke et al., 1998). Expression was also found in embryonic regions including the tail bud, overlapping with that of *Wnt3* (Schubert et al., 2001; Osborne et al., 2009). The present scRNA-seq analysis showed a high level of *Cdx* expression in cells of cluster 5 at stages from late gastrula to early swimming larva, but not in cluster 4 (Figures 4A,C; Supplementary Figure 9A). Expression of *Wnt3* was detected in cells of cluster 5 (Supplementary Figure 8C). These results suggest that cells of cluster 5 occupy the posterior region of the tail bud. In addition to clusters 4 and 5, *Cdx* expression was detected in cells of cluster 0 at late gastrula and cluster 14 at late neurula (Figure 4). Future studies might explore *Cdx* expression in these clusters.

On the other hand, *Notch* expression was very broad and found in cells of most clusters from 0 to 13 (Figure 4; Supplementary Figure 9B). High *Notch* expression was detected in clusters 5, 6, 7, and 13, but only a low level was seen in cluster 4 (Figure 4). In summary, the combinatorial expression of *Brachyury*, *Caudal*, *Notch*, and several *Wnt* genes in clusters 4 and 5 provides additional support for the conclusion that these cells comprise the tail bud in amphioxus neurula.

### Clusters of Cells With Hox Gene Expression

In contrast to the typical cluster of 13 Hox genes in metazoan genomes (Garcia-Fernandez and Holland, 1994), amphioxus duplicated the posterior Hox genes to 15 genes (Minguillon et al., 2005; Supplementary Figure 10). Among them, *Hox1, Hox3*, and *Hox4* are expressed with spatiotemporal collinearity in the developing neural tube of *B. floridae* neurulae (Holland and Garcia-Fernandez, 1996; Wada et al., 1999; Schubert et al., 2004; Pascual-Anaya et al., 2012). *Hox1* is expressed in the developing neural tube of the middle and posterior parts of neurulae, and *Hox1, Hox3*, and *Hox4* show segmental modulation of expression levels, a two-segment phasing of spatial collinearity. Although previous studies focused mainly on *Hox* expression in the developing central nervous system, their results also showed *Hox* expression in the developing mesoderm (somites) as well (Holland and Garcia-Fernandez, 1996; Wada et al., 1999). The present scRNA-seq analysis of *Hox1, Hox3*, and *Hox4* expression profiles demonstrated an additional spatio-expression profile of *Hox* genes, providing new insight to understand evo-devo mechanisms of cephalochordate body plan formation, especially embryonic regions with *Brachyury* expression (Figure 4 and Supplementary Figure 11).

First, in relation to temporal collinearity, initiation of high-level expression of *Hox1, Hox3*, and *Hox4* was detected at early neurula, midneurula, and late neurula, respectively (Figures 4B,C and Supplementary Figure 11). That is, this result confirmed the previous study of temporal collinearity of gene expression: the most-anterior *Hox1* expressed first, followed by anterior *Hox3*, and then middle *Hox4.* Second, in relation to spatial expression, the present analysis showed that expression profiles of *Hox1*, *Hox3*, and *Hox4* very much resemble that of *Bra2*, although the timing of *Bra2* expression was earlier than that of *Hox1*, *Hox3*, and *Hox4* (Figures 4B,C). Specifically, four major clusters with *Hox1, Hox3*, and *Hox4* expression included 4 (tail bud), 5 (tail bud), 8 (somite), and 9 (notochord), in which *Bra2* was specifically expressed (Figure 4). For example, in cluster 5, *Hox1* expression was first detected at late gastrula. It was highest at early neurula and decreased at midneurula, whereas *Hox3* expression was first detected at early neurula, was highest at mid- and late neurula, and decreased at early swimming larva (Figure 4C). *Hox4* expression was detected at late neurula and decreased at early swimming larva (Figure 4C). A similar profile of *Hox* expression was evident in clusters 4, 8, and 9 (Figures 4A–C). This suggests that a combined regulatory network in expression and function of *Brachyury* and *Hox* is involved in the formation of somites, notochord, and tail bud of amphioxus embryos.

### Co-expression of *Bra2* and *Wnt* or *Hox*

**FIGURE 5:**
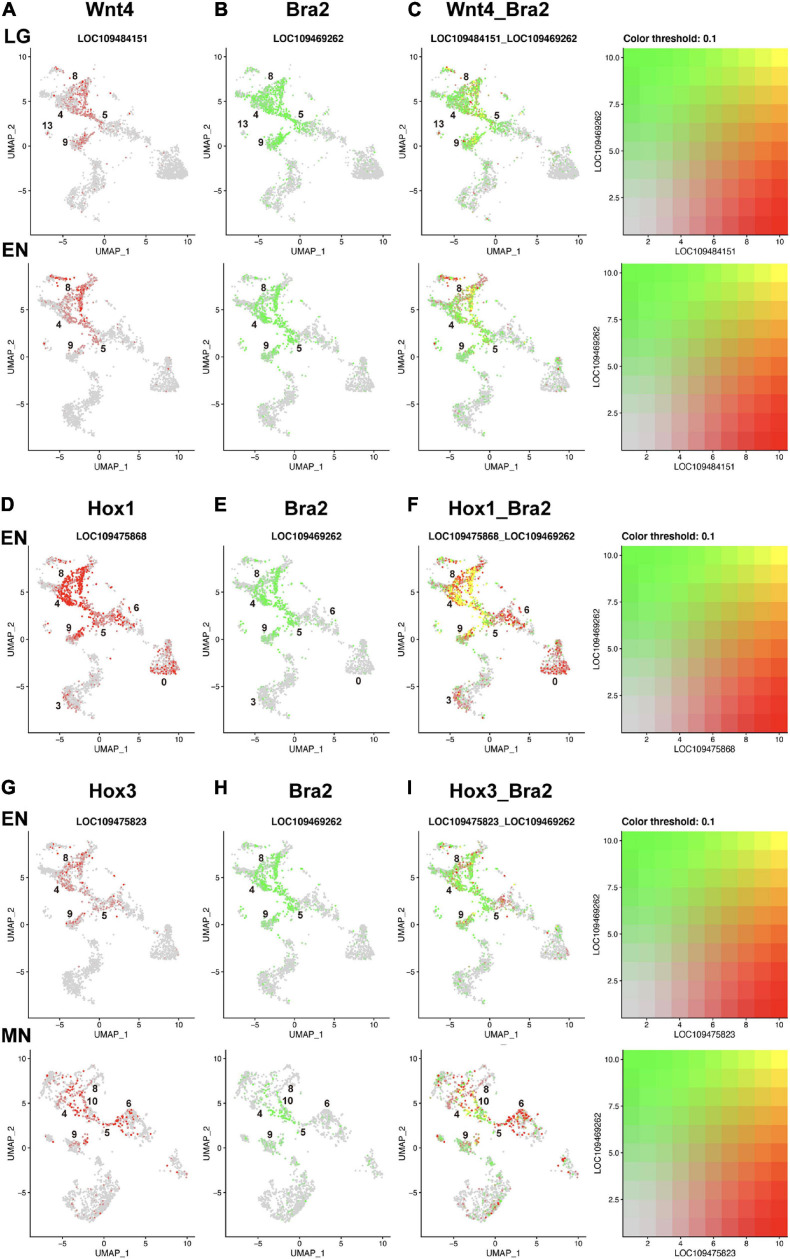
UMAP plots showing co-expression of *Wnt* and *Brachyury*, and *Hox* and *Brachyury* in amphioxus embryos. **(A)** *Wnt4* (red), **(B)** *Bra2* (green), and **(C)** co-expression (yellow) of the two genes at late gastrula (LG) and early neurula (EN) stages. Co-expression is seen in cells of clusters 8, 5, and 9. **(D)** *HoxA1* (red), **(E)** *Bra2* (green), and **(F)** co-expression (yellow) of the two genes at and early neurula (EN) stage. Co-expression is seen in cells of clusters 8, 4, 5, and 9. **(G)** *Hox3* (red), **(H)** *Bra2* (green), and **(I)** co-expression (yellow) of the two genes at early neurula (EN) and midneurula (MN) stages. Co-expression is seen in cells of clusters 8, 4, 5, 9, and 10.

As shown in previous sections, clusters 4, 5, 8 and 9, which have *Bra2* expression, also expressed some *Wnt* and *Hox* genes. Therefore, we examined the grade of co-expression of these genes at the single-cell level by superimposing UMAP plots of the two genes (Figure 5). For example, *Wnt4* was highly expressed in cluster 8 at late gastrula and early neurula, but not in cells of clusters 4, 5, and 9 (Figure 4). Cells of these four clusters were classified to those expressing *Bra2*. Superimposed UMAP plots of the two genes (Figure 5C) indicated co-expression of *Wnt4* and *Bra2* in cells of the blastopore, presumptive somite cells, and presumptive notochord cells at late gastrula and early neurula stages.

Compared to *Wnt* genes, *Hox* genes showed greater co-expression with *Brachyury* in early amphioxus embryos (Figures 5D–F,G–I). *Hox1* showed higher expression in clusters 4, 5, 8, and 9 at early neurula stage (Supplementary Figure 11A), while *Bra2* was expressed in the same clusters at the same stage (Figure 4B). High, single-level co-expression of *Hox1* and *Bra2* was found in many cells of clusters 4, 5, 8, and 9 (Figures 5D–F). Therefore, cells of the blastopore, presumptive somite cells, and presumptive notochord cells at early neurula stages co-expressed *Hox1* and *Bra2.*

Expression *of Hox3* was found in clusters 4, 5, 8, and 9 at neurula stages (Figure 4), and this spatial expression profile was similar to that of *Bra2* (Figure 4). Co-expression of *Hox3* and *Bra2* was found in many cells of clusters 4, 5, 8, and 9 (Figures 5G-I). Therefore, in addition to *Hox1*, cells of the blastopore, presumptive somite cells, and presumptive notochord cells at early neurula stages co-expressed *Hox3* and *Bra2.*

## Discussion

### Gene Expression Profiles of scRNA-seq Analyses and Modes of Embryogenesis

scRNA-seq analyses of gene expression profiles have been reported in early embryonic cells of the sea urchin *Strongylocentrotus* (Foster et al., 2020), the ascidian *Ciona* (Sharman et al., 1999; Cao et al., 2019), zebrafish (Wagner et al., 2018), and *Xenopus* (Briggs et al., 2018). In both echinoderms and ascidians, the number of constituent cells is not very large, approximately 2,000–3,000 cells, even at early larval stages. In addition, embryonic cells are likely specified and differentiated into certain types of organs/tissues in restricted lineages. Accordingly, scRNA-seq analysis resulted in comparatively clear-cut clustering of embryonic cells (Sharman et al., 1999; Cao et al., 2019; Foster et al., 2020). In contrast, embryogenesis of zebrafish and *Xenopus* proceeds by a gradual specification pattern, first regionalizing into three germ layers, ectoderm, endoderm, and mesoderm, and later gradually differentiating to tissues and organs. The number of constituent cells exceeds 20,000 cells at the neurula stage. Therefore, the number of clusters classified by the scRNA-seq method is rather small at early stages, but gradually increases as embryogenesis proceeds (Briggs et al., 2018; Wagner et al., 2018). The mode of amphioxus embryogenesis resembles that of vertebrates (Conklin, 1932; Whittaker, 1997; Holland and Holland, 1998). Therefore, our present scRNA-seq analysis resulted in only 10 clusters at midgastrula stage. In addition, constituent cells of amphioxus embryos increase in number after gastrulation, as in the case of vertebrates. Therefore, the cell sample size examined in this study was depauperate, especially in later stages. We are planning to pursue further analyses to sample a sufficient number of *B. japonicum* embryonic cells in the near future. Nevertheless, this scRNA-seq analysis permitted us to survey cells with *Brachyury* expression.

### Clustering of Amphioxus Embryonic Cells by scRNA-seq

Using a standard bioinformatic protocol for scRNA-seq analysis, we classified embryonic cells of *B. japonicum* into 15 clusters (0 to 14) (Figure 2). Based on results of previous studies that showed specific expression of myogenetic factor genes in the developing paraxial mesoderm or somites, we determined that cluster 8 comprises cells that form somites (Figure 4). Then, we showed that *Brachyury* is expressed in cells of clusters 4, 5, 8, and 9 (Figure 4). Based on data of other genes, we concluded that cluster 9 belongs to the notochord, and clusters 4 and 5 comprise cells of the blastopore and the developing tail bud. These notions were substantiated by simultaneous expression of various transcription factor genes reportedly expressed in the blastopore and notochord (Figure 4). An overall view of clustering by UMAP plots indicates that clusters 4, 5, 8, and 9 have some shared affinity and that they originated from mesoderm (Figure 2).

On the other hand, although UMAP plots (Figure 2) and gene expression profiles (Figure 4) suggest that cluster 0 belongs to ectoderm; clusters 1, 2, 3, 11, and 12 belong to endoderm; and clusters 6, 7, and 10 belong to mesoderm, this study could not unambiguously characterize these clusters, which should be determined in further scRNA-seq analysis. Cluster 13 contains cells with a unique profile of gene expression including *troponin I*, *troponin T*, *gsc*, *hh*, *neterin*, and *Notch* (Figure 4). This cluster also occupies a discrete position in UMAP plots (Figure 2). Characterization of cluster 13 is therefore an interesting subject of future scRNA-seq analysis.

### Development of Embryonic Cells With *Brachyury* Expression

**FIGURE 6:**
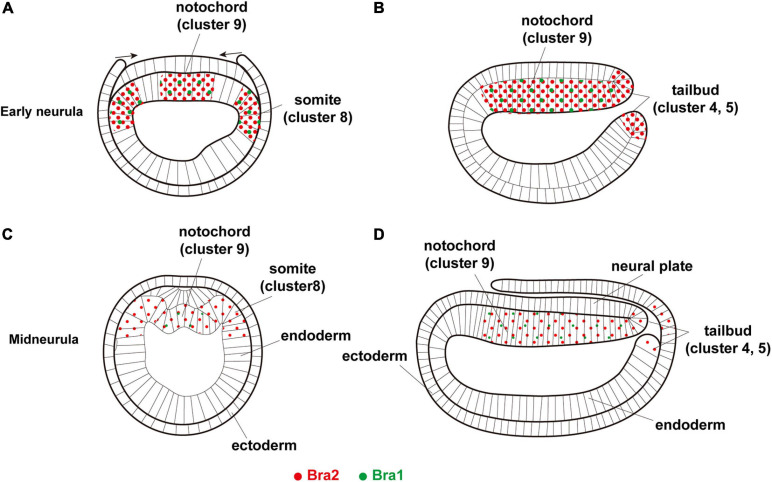
A summary diagram showing expression of two *Brachyury* genes, *Bra1* (green dots) and *Bra2* (red dots), in **(A,B)** early neurula, **(A)** cross section and **(B)** sagittal section, and **(C,D)** midneurula, **(C)** cross section and **(D)** sagittal section. *Bra1* is weakly expressed in the somite (cluster 8) and notochord (cluster 9) at early neurula and becomes undetectable in cluster 8 at midneurula, while a high level of *Bra2* expression is found in the somite (cluster 8), notochord (cluster 9), and tail bud (clusters 4 and 5). Original drawing of embryos by Schubert et al. (2001).

*Brachyury* was duplicated in cephalochordate genomes, independently of *Brachyury* in other deuterostomes (Holland et al., 1995; Inoue et al., 2017). The present scRNA-seq study showed that *Bra2* is expressed at higher levels and in broader embryonic regions than *Bra1* (Figure 6). Since *Bra2*, but not *Bra1*, is expressed in cells of the blastopore (Yuan et al., 2020; this study) and since *Brachyury* expression in the blastopore or archenteron invagination region is a feature shared by most metazoans, *Bra2* is the ancestral *Brachyury* and *Bra1* is a duplicated copy, with more divergent properties, as proposed by previous studies (Tominaga et al., 2018; Yuan et al., 2020). *Bra2* was strongly expressed first in the archenteron invagination region and functions in gastrulation, then in invagination-like movements of cells of the upper archenteron to form the notochord and that of cells of bilateral sides of the archenteron to form somites, and contiguously from the blastopore, in tail bud formation at the posterior-most region of amphioxus embryos. On the other hand, *Bra1* likely retained the potential to form the notochord and somites, serving a supplementary role for *Bra2* in the formation of these organs. These results suggest differences between the two genes in upregulatory transcriptional control, downregulatory transcriptional control of target genes, and combinatorial transcriptional control of other transcription factor genes in the formation of the blastopore, notochord, somites, and tail bud, which should be examined in future studies.

From an evo-devo point of view, the cephalochordate notochord is unique in having muscle-like properties (Ruppert, 1997). Supporting the results of previous studies (Suzuki and Satoh, 2000; Inoue and Satoh, 2018), the present scRNA-seq analysis demonstrated expression of muscle-structural genes in cells of not only cluster 8 (somites), but also cluster 9 (notochord). In other words, during evolution of cephalochordates, *Brachyury* recruited muscle structural genes under its expressional control. Even more intriguing is the complexity of muscle structural genes. Many muscle structural genes are expressed in the developing somites, while some muscle structural genes are also expressed in the developing notochord, and some are expressed in both somites and notochord (Figure 4). Therefore, the muscle-like properties of the amphioxus notochord did not appear by simply copying somites. Future studies should explore molecular and cellular mechanisms involved in this unique muscle gene array of the amphioxus notochord.

The present study also revealed differences in the temporal specification of the notochord and somites. Notochord cluster 9 appeared at the midgastrula stage, suggesting that specification of cells that give rise to the notochord have already occurred (Figures 2, 4B). On the other hand, somite cluster 8 was not formed at the midgastrula stage and first appeared at the late gastrula stage (Figures 2, 4B). Although further studies are required to clarify this issue, these results suggest that the notochord specification occurs earlier than somite specification in amphioxus gastrulae.

### Expression of Transcription Factor Genes in Cells With *Brachyury* Expression

Cephalochordates are a research target to understand the origin and evolution of chordates (Holland et al., 2015; Satoh, 2016; Gee, 2018). To date, a large number of evo-devo studies have cloned cDNA of genes for major transcription factors and signaling molecules and extensively examined their spatiotemporal expression profiles by WMISH (Holland et al., 1998; Jackman et al., 2000; Meulemans and Bronner-Fraser, 2002; Schubert et al., 2003; Yu et al., 2007; Wu et al., 2011; Wang et al., 2016). Many such genes show overlapping expression with other genes due to the gradual specification mode of amphioxus embryos. Because *Bra2* is expressed broadly in the embryonic blastopore, somites, notochord, and tail bud, cells with *Bra2* expression simultaneously expressed other transcription factor genes. Here, we discuss only simultaneous expression of *Bra2* and Hox cluster genes, including *Hox1, Hox3*, and *Hox4* in cells of clusters 4, 5, 8, and 9. This was clearly shown in UMAP plot figures (Figure 5). The presence of cells with co-expression of *Bra2* and *Hox1, Hox3*, and *Hox4* has not been reported previously. Although details should be explored in future studies, the similarity of expression profiles in cells of clusters 4 (blastopore and tail bud), 5 (blastopore and tail bud), 8 (somites), and 9 (notochord) suggests coordinated roles of *Bra2* and *Hox* in the development of these major organs during amphioxus body plan formation.

Contiguous with the blastopore, the tail bud contains cells with various properties, including cells of the nervous system, mesoderm, and endoderm. As reported in *Xenopus* (Gentsch et al., 2013), *Bra2* expression in the nervous system might be involved in joint regulation of embryonic neuro-mesodermal bipotency in amphibian embryos as well. *Brachyury, Wnt*, genes, *caudal*, and *Notch* are expressed in the tail bud in a coordinated fashion (Holland, 2002). This scRNA-seq analysis showed simultaneous and preferential expression of *Brachyury*, multiple *Wnt* genes, and *caudal* in cells of the tail bud, while *Notch* expression was found in the tail bud, but was rather broadly seen in other embryonic regions as well (Figure 4). In addition, this scRNA-seq analysis indicates that clusters 4 and 5 contain cells with simultaneous expression of these genes, suggesting the presence of two different cell types in the developing tail bud. Although we could not distinguish different properties of cells in clusters 4 and 5 at present, this may be an important focus of future studies, because the tail bud is an evolutionary novelty that occurred in chordate embryos and is profoundly involved in the establishment of the chordate-specific body plan.

## Materials and Methods

### Branchiostoma japonicum

*Branchiostoma japonicum* has been maintained from generation to generation for more than 10 years, first in an aquaculture system at Kumamoto University (Yasui et al., 2013) and then at Tateyama Marine Laboratory of Ochanomizu University in Japan. Adults with mature eggs or sperm were transferred to the Marine Genomics laboratory of Okinawa Institute of Science and Technology Graduate University (OIST). Spawning was induced by controlling temperature and light. Naturally spawned eggs and sperm were mixed to achieve fertilization. All embryos used in this study resulted from mating of one male and one female. Embryos were cultured in Petri dishes filled with filtered natural seawater (pore size, 1 μm). We collected embryos and larvae at six different stages including midgastrula (G3 stage), late gastrula (G5 stage), early neurula (N0 stage), midneurula (N3 stage), late neurula (N5 stage), and early swimming larva (T1 stage). Staging was based on embryonic gross morphology, referring to descriptions of developmental stages of *B. floridae* (Whittaker, 1997) and *B. lanceolatum* (Carvalho et al., 2021).

### Embryonic Cell Dissociation and Single-Cell RNA Sequencing

Embryos or larvae at appropriate stages were collected and washed with 1.2 M glycine solution three or four times. For prehatching stages, the fertilization membrane was removed. Then, embryos or larvae were pipetted in 1.2 M glycine solution on ice until cells were completely dissociated. Dissociated cells were counted using hemocytometers (C-chip, NanoEnTek DHC-N01) and diluted with 1.2 M glycine solution to reach an appropriate concentration for the scRNA-seq protocol. Single-cell encapsulation, cDNA synthesis, and library preparation were performed using Chromium Single-Cell 3′ Reagent Kit v3 Chemistry. Libraries were sequenced on an Illumina NovaSeq. Sequencing reads of the six embryonic stages are summarized in Supplementary Table 1. Except for midgastrula (350 million reads), more than 600 million reads were obtained for each stage (Supplementary Table 1).

### Clustering of Cells That Differed in Gene Expression

Single-cell unique molecular identifier (UMI) counting (counting of unique barcodes given to individual transcript molecules) was performed using Cell Ranger Single-Cell Software Suite 4.0.0 from 10 × Genomics. We aligned reads to the *B. belcheri* assembly Haploidv18h27 (GCF_001625305.1) using CellRanger. CellRanger gene expression matrices were further analyzed using the R package Seurat v 4.0.1 (Butler et al., 2018; Stuart et al., 2019; Hao et al., 2020).

Genomes of *B. floridae* (Putnam et al., 2008; Simakov et al., 2020) and *B. belcheri* (Huang et al., 2014) have been decoded, but the *B. japonicum* genome has not been sequenced yet. However, previously, *B. japonicum* was called *B. belcheri*, and taxonomically reclassified as a new species independent of *B. belcheri* approximately 15 years ago (Zhang et al., 2006). Many previous cDNA cloning studies using *B. belcheri*, especially those done by Japanese researchers, likely used this name. Therefore, we here used the *B. belcheri* gene models as references for RNA read annotation.

The number of RNA-seq reads and cells examined at each stage are shown in Supplementary Tables 1,2. Supplementary Table 2 also shows the number of cells examined in each of the 15 clusters. Between 500 and 2500 cells were included in downstream analysis. Individual datasets were normalized by scaling gene expression in each cell by total gene expression, followed by log transformation. The top 2000 highly variable genes across datasets were then used to integrate datasets. Individual time-point datasets were integrated (employing the Seurat v4 pipeline) to identify conserved cell populations across datasets. This technique involves pairwise comparisons of individual cells across multiple datasets followed by hierarchical clustering. UMAP is a dimensional reduction technique. Projection and clustering analysis for visualization of integrated data was conducted using 36 parameter dimensions and a resolution of 0.5 (Stuart et al., 2019). Thirty-six dimensions (i.e., inclusion of 36 principal components) were used in consideration of principal component heatmaps, which show sources of heterogeneity in a dataset, and the ElbowPlot function, which depicts the number of principal components that include variance present in the data. The dataset was also visualized at a resolution of 3 to provide an example of how additional cell states may be revealed, including subtypes of states seen and identified at a resolution 0.5. No one resolution setting is optimal for all clusters, but these disparate settings are intended to assist the reader in data interpretation and identification of candidate genes. The resolution parameter of the FindClusters function can be modulated to show more or fewer clusters and a series of different resolutions can be tested before choosing a value that is appropriate for the biological context of an experiment.

### Molecular Phylogeny Analysis

Phylogenetic relationships of family member genes for MFs, T-box, Wnt, and Hox in amphioxus genomes were examined using ORTHOSCOPE (Inoue and Satoh, 2018)^1^. In short, BLAST hit sequences were screened using an *E*-value cutoff of < 10^–3^ and the top five BLAST hits were selected for subsequent analyses. Phylogenetic trees were estimated using the NJ method with the first and second codon positions. To evaluate robustness of internal branches, 100 bootstrap replications were calculated for each dataset.

## Data Availability Statement

The datasets presented in this study can be found at the NCBI BioProject (http://www.ncbi.nlm.nih.gov/bioproject/) under BioProject accession PRJDB10575 and BioSample accessions SAMD00294414–SAMD00294419.

## Author Contributions

NS, HT, and KN designed the study. MK cultured *Branchiostoma japonicum.* HT and KN carried out molecular lab work. KH and JI analyzed data, carried out statistical analysis, and imaged results. NS wrote the first draft of the manuscript. All authors commented on and approved the final manuscript.

## Conflict of Interest

The authors declare that the research was conducted in the absence of any commercial or financial relationships that could be construed as a potential conflict of interest.

## Footnotes

1 https://www.orthoscope.jp

## References

[B1] Aase-Remedios M. E. Coll-Llado C. Ferrie D. E. (2020). More than one-to-four via 2R: evidence of an independent amphioxus expansion and two-gene ancestral vertebrate state for MyoD-related myogenic regulatory factors (MRFs). *Mol. Biol. Evol.* 37 2966–2982. 10.1093/molbev/msaa147 32520990 PMC7530620

[B2] Araki I. Terazawa K. Satoh N. (1996). Dulication of an amphioxus myogentic bHLH gene is independent of vertebrate myogenic bHLH gene duplication. *Gene* 171 231–236. 10.1016/0378-1119(96)00174-6 8666278

[B3] Bertrand S. Escriva H. (2011). Evolutionary crossroads in developmental biology: amphioxus. *Development* 138 4819–4830. 10.1242/dev.066720 22028023

[B4] Bourlat S. J. Juliusdottir T. Lowe C. J. Freeman R. Aronowicz J. (2006). Deuterostome phylogeny reveals monophyletic chordates and the new phylum Xenoturbellida. *Nature* 444 85–88. 10.1038/nature05241 17051155

[B5] Briggs J. A. Weinreb C. Wagner D. E. Medason S. Peshkin L. Kirschner M. W. (2018). The dynamics of gene expression in vertebrate embryogenesis at single-cell resolution. *Science* 360:eaar5780. 10.1126/science.aar5780 29700227 PMC6038144

[B6] Brooke N. M. Garcia-Fernàndez J. Holland P. W. H. (1998). The ParaHox gene cluster is anevolutionary sister of the Hox gene cluster. *Nature* 392 920–922. 10.1038/31933 9582071

[B7] Butler A. Hoffman P. Smibert P. Papalexi E. Satija R. (2018). Integrating single-cell transcriptomic data across different conditions, technologies, and species. *Nat. Biotechnol.* 36 411–420. 10.1038/nbt.4096 29608179 PMC6700744

[B8] Cao C. Lemaire I. A. Wang W. Yoon P. H. Choi Y. A. Parsons L. R. (2019). Comprehensive single-cell transcriptome lineages of a proto-vertebrate. *Nature* 571 349–354. 10.1038/s41586-019-1385-y 31292549 PMC6978789

[B9] Carvalho J. E. Lahaye F. Yong L. W. Croce J. C. Escrivá H. Yu J.-K. (2021). An updated staging system for cephalochordate development: one table suits them all. *bioRxiv* [Preprint] 10.1101/2020.05.26.112193 PMC8174843 34095136

[B10] Conklin E. G. (1932). The embryology of amphioxus. *J. Morph.* 54 69–151. 10.1002/jmor.1050540103

[B11] Delsuc F. Brinkmann H. Chourrout D. Philippe H. (2006). Tunicates and not cephalochordates are the closest living relatives of vertebrates. *Nature* 439 965–968. 10.1038/nature04336 16495997

[B12] Foster S. Oulhen N. Wessel G. (2020). A single cell RNA sequencing resource for early sea urchin development. *Development* 147:dev191528. 10.1242/dev.191528 PMC7502599 32816969

[B13] Garcia-Fernandez J. Holland P. W. H. (1994). Archetypal organization of the amphixus Hox gene cluster. *Nature* 370 563–566. 10.1038/370563a0 7914353

[B14] Gee H. (2018). *Across the Bridge: Understanding the Origin of the Vertebrates.* Chicago, IL: The University of Chicago Press.

[B15] Gentsch G. E. Owens N. D. L. Martin S. R. Piccinelli P. Faial T. Trotter M. W. B. (2013). In vivo T-box transcription factor profiling reveals joint regulation of embryonic neuromesodermal bipotency. *Cell Rep.* 4 1185–1196. 10.1016/j.celrep.2013.08.012 24055059 PMC3791401

[B16] Gross J. M. McClay D. R. (2001). The role of Brachyury (T) during gastrulation movements in the sea urchin *Lytechinus variegatus*. *Dev. Biol.* 239 132–147. 10.1006/dbio.2001.0426 11784024

[B17] Hao Y. Hao S. Andersen-Nissen E. Mauck W. M. III Zheng S. Butler A. (2020). Integrated analysis of multimodal single-cell data. *bioRxiv* [Preprint] 10.1101/2020.10.12.33533 PMC8238499 34062119

[B18] Hirakow R. Kajita N. (1994). Electron miscroscopic study of development of amphixus. *Acta Anat. Nippon.* 69 1–13. 8178614

[B19] Holland L. Z. (2002). Heads or tails? Amphioxus and the evolution of anterior-posterior patterning in deuterostomes. *Dev. Biol.* 241 209–228. 10.1006/dbio.2001.0503 11784106

[B20] Holland L. Z. Holland N. D. Schubert M. (2000). Developmental expression of *AmphiWnt1*, an amphioxus gene in the Wnt1/wingless subfamily. *Dev. Genes Evol.* 10 522–524. 10.1007/s004270000089 11180802

[B21] Holland L. Z. Venkatesh T. V. Gorlin A. Bodmer R. Holland V. D. (1998). Characterization and developmental expression of AmphiNk2.2, an NK2 class homeobox gene from amphioxus (Phylum Chordata; Subphylum Cephalochordata). *Dev. Genes Evol.* 208 100–105. 10.1007/s004270050159 9569351

[B22] Holland N. D. Holland L. (1998). Developmental gene expression in amphioxus: new insight into the evolution. *Am. Zool.* 38 647–658.

[B23] Holland N. D. Holland N. D. Holland P. W. (2015). Scenarios for the making of vertebrates. *Nature* 520 450–455. 10.1038/nature14433 25903626

[B24] Holland P. W. H. Garcia-Fernandez J. (1996). Hox genes and chordate evolution. *Dev. Biol.* 173 382–395. 8605999 10.1006/dbio.1996.0034

[B25] Holland P. W. Koschorz B. Holland L. Z. Herrmann B. G. (1995). Conservation of Brachyury (T) genes in amphioxus and vertebrates: developmental and evolutionary implications. *Development* 121 4283–4291. 10.1242/dev.121.12.4283 8575328

[B26] Huang S. Chen Z. Yan X. Yu T. Huang G. Yan Q. (2014). Decelerated genome evolution in modern vertebrates revealed by analysis of multiple lancelet genomes. *Nat. Commun.* 5:5896. 10.1038/ncomms6896 25523484 PMC4284660

[B27] Imai K. Levine M. Satoh N. Satou Y. (2006). Regulatory blueprint for a chordate embryo. *Science* 312 1183–1187. 10.1126/science.1123404 16728634

[B28] Inoue J. Satoh N. (2018). Deuterostome genomics: lineage-specific protein expansions that enabled chordate muscle evolution. *Mol. Biol. Evol.* 35 914–924. 10.1093/molbev/msy002 29319812 PMC5888912

[B29] Inoue J. Yasuoka Y. Takahashi H. Satoh N. (2017). The chordate ancestor possessed a single copy of Brachyury gene for notochord acquisition. *Zool. Lett.* 3:4. 10.1186/s40851-017-0064-9 28344820 PMC5363035

[B30] Jackman W. R. Langeland J. A. Kimmel C. B. (2000). Islet reveals segmentation in the amphioxus hindbrain homolog. *Dev. Biol.* 220 16–26. 10.1006/dbio.2000.9630 10720427

[B31] Jiang D. Smith W. C. (2007). Ascidian notochord morphogenesis. *Dev. Dyn.* 236 1748–1757. 10.1002/dvdy.21184 17497687 PMC2922061

[B32] Lowe C. J. Clark D. N. Medeiros D. M. Rokhsar D. S. Gerhart J. (2015). The deuterostome context of chordate origins. *Nature* 520 456–465. 10.1038/nature14434 25903627

[B33] Marletaz F. Firbas P. N. Maeso I. Tena J. J. Bogdanovic O. Perry M. (2018). Amphioxus functional genomics and the origins of vertebrate gene duplication. *Nature* 564 64–70. 30464347 10.1038/s41586-018-0734-6 PMC6292497

[B34] Meulemans D. Bronner-Fraser M. (2002). Amphioxus and lamprey AP-2 genes: implication for neural crest evolution and migration patterns. *Development* 129 4953–4962. 10.1242/dev.129.21.4953 12397104

[B35] Minguillon C. Gardenyes J. Serra E. Castro L. F. Hill-Force A. Holland P. W. H. (2005). No more than 14: the endo of amphioxus Hox cluster. *Int. J. Biol. Sci.* 1 19–23. 10.7150/ijbs.1.19 15951846 PMC1140354

[B36] Munro E. M. Odell G. M. (2002). Polarized basolateral cell motility underlies invagination and convergent extension of the ascidian notochord. *Development* 129 13–24. 10.1242/dev.129.1.13 11782397

[B37] Neidert A. H. Panopoulou G. Langeland J. A. (2008). Amphioxus *goosecoid* and the evolution of the head organizer and prechordal plate. *Evol. Dev.* 2 303–310. 10.1046/j.1525-142x.2000.00073.x 11256375

[B38] Oliveri P. Walton K. D. Davidson E. H. McClay D. R. (2006). Repression of mesodermal fate by foxa, a key endoderm regulator of the sea urchin embryo. *Development* 133 4173–4181. 10.1242/dev.02577 17038513

[B39] Onai T. Aramaki T. Inomata H. Hirai T. Kuratani S. (2015). Ancestral mesodermal reorganization and evolution of the vertebrate head. *Zool. Lett.* 1:29. 10.1186/s40851-015-0030-3 26605074 PMC4657371

[B40] Ono H. Koop D. Holland L. Z. (2018). Nodal and Hedgehog synergize in gill slit formation during development of the cephalochordate *Branchiostoma floridae*. *Development* 145:dev162586. 10.1242/dev.162586 29980563

[B41] Osborne P. W. Benoit G. Laudet V. Schubert M. Ferrier D. E. K. (2009). Differential regulation of ParaHox genes by retinoic acid in the invertebrate chordate amphioxus (*Branchiostoma floridae*). *Dev. Biol.* 327 252–262. 10.1016/j.ydbio.2008.11.027 19103191

[B42] Pascual-Anaya J. Adachi N. Alvarez S. Kuratani S. D’Aniello S. Garcia-Fernàndez J. (2012). Broken colinearilty of the amphioxius Hox cluster. *EvoDevo* 3:28. 10.1186/2041-9139-3-28 23198682 PMC3534614

[B43] Petillon Y. L. Luxardi G. Scerbo P. Cibois M. Leon A. Subirana L. (2017). Nodal-activin pathway is a conserved neural induction signal in chordates. *Nat. Ecol. Evol*. 1 1192–1200. 10.1038/s41559-017-0226-3 28782045 PMC5540175

[B44] Putnam N. H. Butts T. Ferrier D. E. Furlong R. F. Hellsten U. Kawashima T. (2008). The amphioxus genome and the evolution of the chordate karyotype. *Nature* 453 1064–1071. 18563158 10.1038/nature06967

[B45] Ruppert E. E. (1997). “Cephalochordata (Acrania),” in *Microscopic Anatomy of Invertebrates*, ed. Harrison F. W. (New York, NY: Willey-Liss).

[B46] Satoh G. Takeuchi J. K. Yasui K. Tagawa K. Saiga H. Zhang P. (2002). *Amphi-Eomes/Tbr1*: an amphioxus cognate of vertebrate *Eomesodermi*n and *Tbrain1* genes whose expression reveals evolutionarily distinct domain in amphioxus development. *J. Exp. Zool.* 294 136–145. 10.1002/jez.10149 12210114

[B47] Satoh N. (2016). *Chordate Origins and Evolution: The Molecular Evolutionary Road to Vertebrates.* San Diego, CA: Academic Press.

[B48] Satoh N. Tagawa K. Takahashi H. (2012). How was the notochord born? *Evol. Dev.* 14 56–75. 10.1111/j.1525-142x.2011.00522.x 23016975

[B49] Schubert M. Holland L. Z. Panopoulou G. D. Lehrach H. Holland N. D. (2000). Characterization of amphioxus *AmphiWnt8*: insights into the evolution of patterning of the embryonic dorsoventral axis. *Evol. Dev.* 2 85–92. 10.1046/j.1525-142x.2000.00047.x 11258394

[B50] Schubert M. Holland L. Z. Stokes M. D. Holland N. D. (2001). Three amphioxus Wnt genes (AmphiWnt3, AmphiWnt5, and AmphiWnt6) associated with the tail bud: the evolution of somitogenesis in chordates. *Dev. Biol.* 240 262–273. 10.1006/dbio.2001.0460 11784062

[B51] Schubert M. Holland N. D. Escriva H. Holland L. Z. Laudet V. (2004). Retinoic acid influences anteroposterior positioning of epidermal sensory neurons and their gene expression in a developing chordate (amphioxus). *Proc. Natl. Acad. Sci. U.S.A.* 101 10320–10325. 10.1073/pnas.0403216101 15226493 PMC478570

[B52] Schubert M. Muelemans D. Bronner-Fraser M. Holland L. Z. Holland N. D. (2003). Differential mesodermal expression of two *MyoD* family members (*AmphiMRF1* and *AmphiMRF2*). *Gene Expr. Patterns* 3 199–202. 10.1016/s1567-133x(02)00099-6 12711549

[B77] Sharma S. Wang W. Stolfi A. (2019). Single-cell transcriptome profiling of the *Ciona* larval brain. *Dev. Biol.* 448 226–236. 10.1016/j.ydbio.2018.09.023 30392840 PMC6487232

[B53] Sharman A. C. Shimeld S. Holland P. W. H. (1999). An amphioxus Msx gene expressed predominantly in the dorsal neural tube. *Dev. Genes Evol.* 209 260–263. 10.1007/s004270050251 10079370

[B54] Shimeld S. M. (1997). Characterization of amphioxus HNF-3 genes: conserved expression in the notochord and floor plate. *Dev. Biol.* 183 74–85. 10.1006/dbio.1996.8481 9119116

[B55] Shimeld S. M. (1999). The evolution of the hedgehog gene family in chordates: insights from amphioxus hedgehog. *Dev. Genes Evol.* 209 40–47. 10.1007/s004270050225 9914417

[B56] Shimeld S. M. (2000). An amphioxus netrin gene is expressed in midline structres during embryonic and larval development. *Dev. Genes Evol.* 210 337–344. 10.1007/s004270000073 11180840

[B57] Simakov O. Marlétaz F. Yue J. X. O’Connell B. Jenkins J. Brandt A. (2020). Deeply conserved synteny resolves early events in vertebrate evolution. *Nat. Ecol. Evol.* 4 820–830. 10.1038/s41559-020-1156-z 32313176 PMC7269912

[B58] Stuart T. Satija R. (2019). Integrative single-cell analysis. *Nat. Rev. Genet.* 20 257–272. 30696980 10.1038/s41576-019-0093-7

[B59] Stuart T. Butler A. Hoffman P. Hafemeister C. Papalexi E. Mauck W. M. III (2019). Comprehensive integration of single-cell data. *Cell* 177 1888–1902.e21. 31178118 10.1016/j.cell.2019.05.031 PMC6687398

[B60] Suzuki M. M. Satoh N. (2000). Genes expressed in the amphioxus notochord revealed by EST analysis. *Dev. Biol.* 224 168–177. 10.1006/dbio.2000.9796 10926757

[B61] Tagawa K. Humphreys T. Satoh N. (1998). Novel pattern of *Brachyury* gene expression in hemichordate embryos. *Mech. Dev.* 75 139–143. 10.1016/s0925-4773(98)00078-1 9739128

[B62] Tan X. Zhang P. J. Du S. J. (2014). Evolutionary aspects of a new MyoD gene in amphioxus (*Branchiostoma belcheri*) and its promoter specificity in skeletal and cardiac muscle. *Biologia* 69 1210–1224. 10.2478/s11756-014-0427-z

[B63] Terazawa K. Satoh N. (1997). Formation of the chordamesoderm in the amphioxus embryo: analysis with *Brachyur*y and *fork head/HNF-3* genes. *Dev. Genes Evol.* 207 1–11. 10.1007/s004270050086 20607475

[B64] Tominaga H. Satoh N. Ueno N. Takahashi H. (2018). Enhancer activities of amphioxus Brachyury genes in embryos of the ascidian, *Ciona intestinalis*. *Genesis* 58:e23240. 10.1002/dvg.23240 30113767

[B65] Urano A. Suzuki M. Zhang P. Satoh N. Satoh K. (2003). Expression of muscle-related genes and two MyoD genes during amphioxus notochord development. *Evol. Dev.* 5 447–458. 10.1046/j.1525-142x.2003.03051.x 12950624

[B66] Wada Y. Garcia-Fernandez J. Holland P. W. H. (1999). Colinear and segmental expression of amphioxus Hox genes. *Dev. Biol.* 213 131–141. 10.1006/dbio.1999.9369 10452851

[B67] Wagner D. E. Weinreb C. Collins Z. M. Briggs J. A. Megason S. G. Klein A. M. (2018). Single-cell mapping of gene expression landscapes and lineage in the zebrafish embryo. *Science* 360 981–987. 10.1126/science.aar4362 29700229 PMC6083445

[B68] Wang J. Li G. Qian G.-H. Wang Y.-Q. (2016). Expression analysis of eight amphioxus genes involved in the Wnt/beta-catenin signaling pathway. *Zool. Res.* 37 136–143. 10.13918/j.issn.2095-8137.2016.3.136 PMC4914576 27265651

[B69] Whittaker J. R. (1997). “Cephalochordates, the lancelets,” in *Embryology: Constructing the Organism*, eds Gilbert S. F. Raunio A. M. (Sunderland, MA: Sinauer).

[B70] Wu H.-R. Chen Y.-T. Su Y.-H. Luo Y. J. Holland L. Z. Yu J.-K. (2011). Asymmetric localization of germline markers Vasa and Nanos during early development in the amphioxus *Branchiostoma floridae*. *Dev. Biol.* 353 147–159. 10.1016/j.ydbio.2011.02.014 21354126

[B71] Yasui K. Igawa T. Kaji T. Henmi Y. (2013). Stable aquaculture of the Japanese lancelet *Branchiostoma* japonicum for 7 years. *J. Exp. Zool. B Mol. Dev. Evol.* 320B 538–547. 10.1002/jez.b.22540 24006276

[B72] Yu J.-K. Mazet F. Chen Y.-T. Haung S.-W. Jung K.-C. Shimeld S. M. (2008). The *Fox* genes of *Branchiostoma floridae*. *Dev. Genes Evol.* 218 629–638. 18773219 10.1007/s00427-008-0229-9

[B73] Yu J.-K. Satou Y. Holland N. D. Shin-I T. Kohara Y. Satoh N. (2007). Axial patterning in cephalochordates and the evolution of the organizer. *Nature* 445 613–617. 10.1038/nature05472 17237766

[B74] Yuan L. Wang Y. Li G. (2020). Differential expression pattern of two Brachyury genes in amphioxus embryos. *Gene Expr. Patterns* 38:119152. 10.1016/j.gep.2020.119152 33115671

[B75] Zhang Q.-J. Zhong J. Fang S.-H. Wang Y.-Q. (2006). *Branchiostoma japonicum* and *B. belcheri* are distinct lancelets (Cephalochordata) in Xiamen waters in China. *Zool. Sci.* 23 573–579. 10.2108/zsj.23.573 16849846

[B76] Zhu X. Shi C. Zhong Y. Liu X. Yan Q. Wu X. (2020). Cilia-driven asymmetric Hedgehog signalling determines the amphioxus left-right axis by controlling Dand5 expression. *Development* 147:dev182469. 10.1242/dev.182469 31826864

