# Supplementary material for: A Preliminary Single-Cell RNA-Seq Analysis of Embryonic Cells That Express Brachyury in the Amphioxus, Branchiostoma japonicum
Source: Front Cell Dev Biol. 2021 Jul 15;9:696875. doi: 10.3389/fcell.2021.696875 (PMC8321703; doi:10.3389/fcell.2021.696875)
Supplement: Supplementary file 1 [file Table_1.PDF]

Supplementary Table S1 Sequence results of *Branchiostoma japonicum* scRNA-seq

Developmental Stage	R1 length* (bp)	R2 length** (bp)	Number of R2 reads	Number of detected cells	Mean reads per cell
mid Gastrula	28	91	352,176,495	2250	2363.125
late Gastrula	28	91	660,981,365	3173	1031.332
early neurula	28	91	681,254,298	2622	888.3268
mid neurula	28	91	660,749,485	2253	665.8744
late neurula	28	91	619,103,669	2023	688.5571
swimming larva	28	91	658,772,670	1695	352.7375

*Sequence for Cell barcode and UMI

**Sequence for sample
