# Supplementary material for: A Preliminary Single-Cell RNA-Seq Analysis of Embryonic Cells That Express Brachyury in the Amphioxus, Branchiostoma japonicum
Source: Front Cell Dev Biol. 2021 Jul 15;9:696875. doi: 10.3389/fcell.2021.696875 (PMC8321703; doi:10.3389/fcell.2021.696875)
Supplement: Supplementary file 2 [file Table_2.PDF]

Supplementary Table S2. Number of cells obtained to use scRNA-seq analysis

Cluster	0	1	2	3	4	5	6	7	8	9	10	11	12	13	14	total
All_stages	1955	1725	1610	1543	1414	1276	855	721	682	665	585	490	238	210	47	14016
MG	462	1	1304	3	2	454	0	0	0	19	3	1	1	0	0	2250
LG	787	190	150	228	539	309	103	71	202	249	143	128	65	9	0	3173
EN	459	219	49	380	419	269	150	86	235	163	49	66	65	13	0	2622
MN	83	450	19	190	166	146	377	240	144	170	13	110	31	98	16	2253
LN	4	538	14	381	231	68	160	197	79	55	5	132	45	87	27	2023
ESL	160	327	74	361	57	30	65	127	22	9	372	53	31	3	4	1695
