# Supplementary material for: A Preliminary Single-Cell RNA-Seq Analysis of Embryonic Cells That Express Brachyury in the Amphioxus, Branchiostoma japonicum
Source: Front Cell Dev Biol. 2021 Jul 15;9:696875. doi: 10.3389/fcell.2021.696875 (PMC8321703; doi:10.3389/fcell.2021.696875)
Supplement: Supplementary file 3 [file Data_Sheet_1.PDF]

Supplementary Figure 1

(A) Myogenic genes

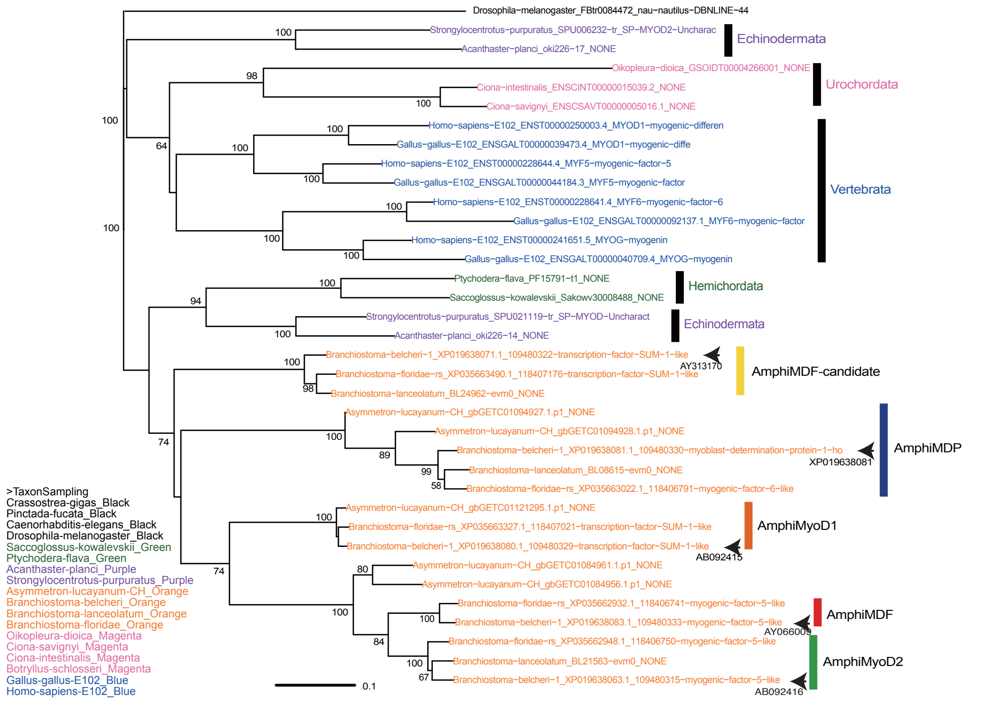

(B)

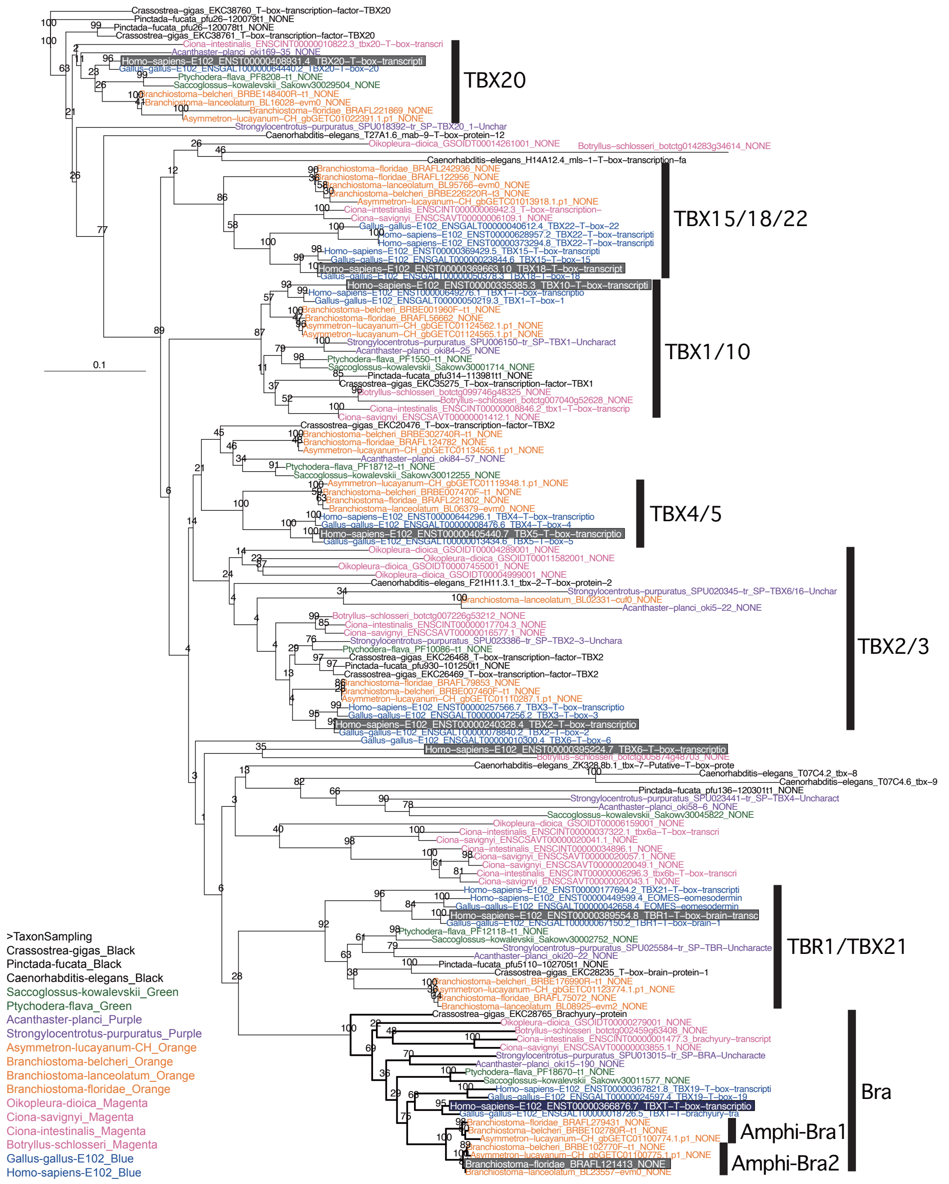

Supplementary Figure 4. Molecular phylogeny of four myogenic factor genes and T-box family genes in amphioxus. **(A)** Myogenic factor genes in amphioxus genomes. **(B)** Brachyury genes in amphioxus genomes.
