# Supplementary figures and images for: A Preliminary Single-Cell RNA-Seq Analysis of Embryonic Cells That Express Brachyury in the Amphioxus, Branchiostoma japonicum
Source: Front Cell Dev Biol. 2021 Jul 15;9:696875. doi: 10.3389/fcell.2021.696875 (PMC8321703; doi:10.3389/fcell.2021.696875)

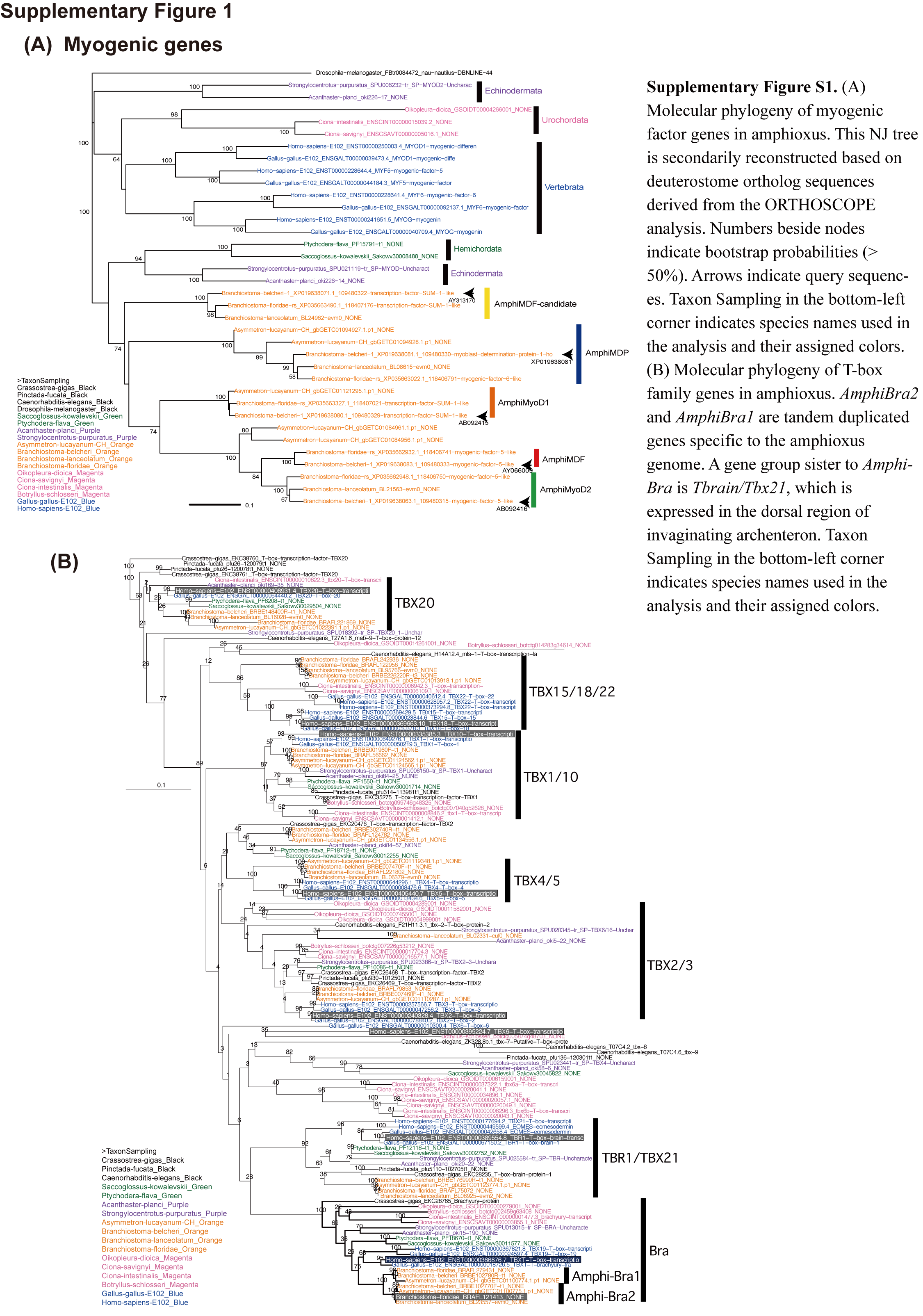

Supplement: Supplementary file 4 [file Image_1.tif]

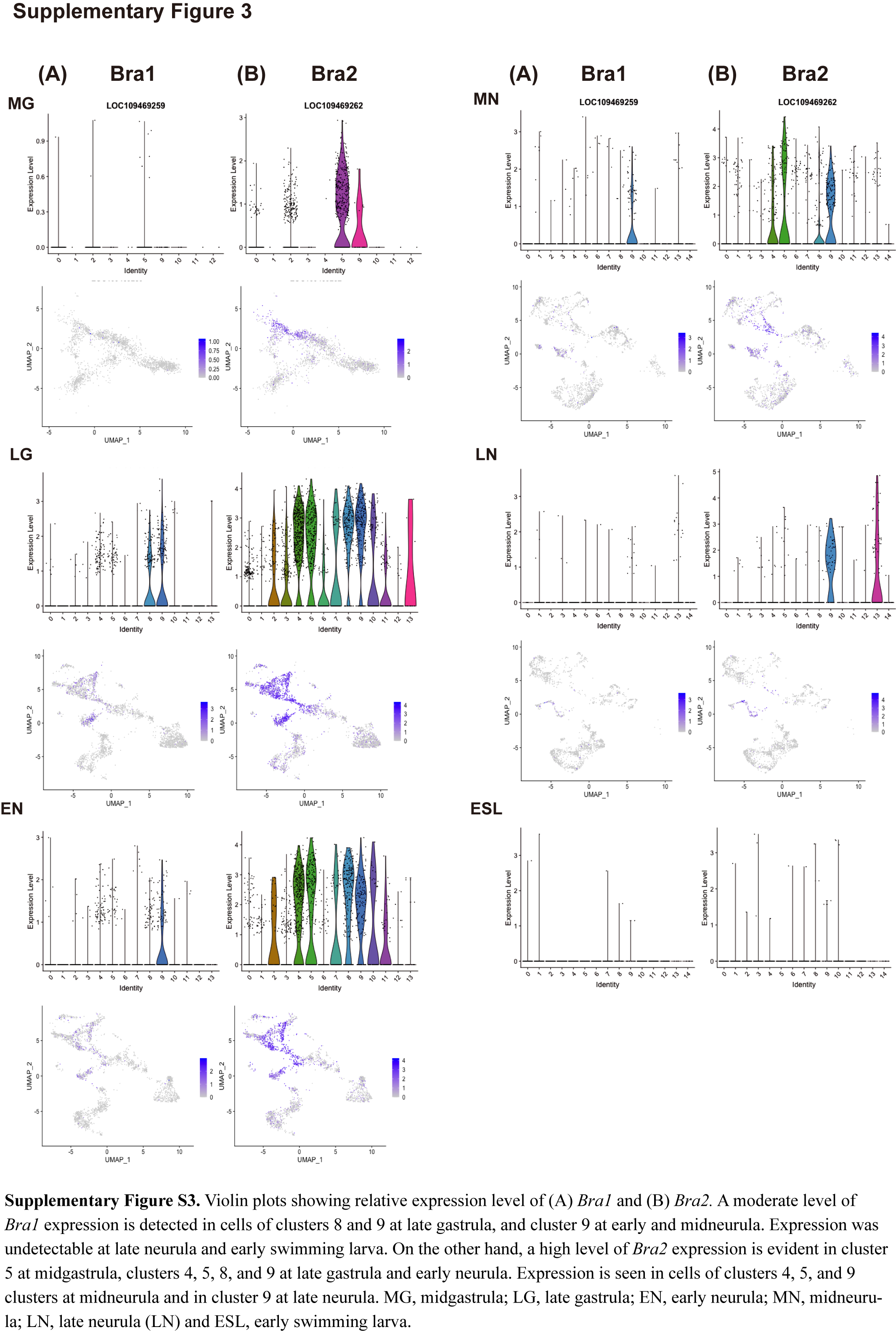

Supplement: Supplementary file 5 [file Image_2.TIF]

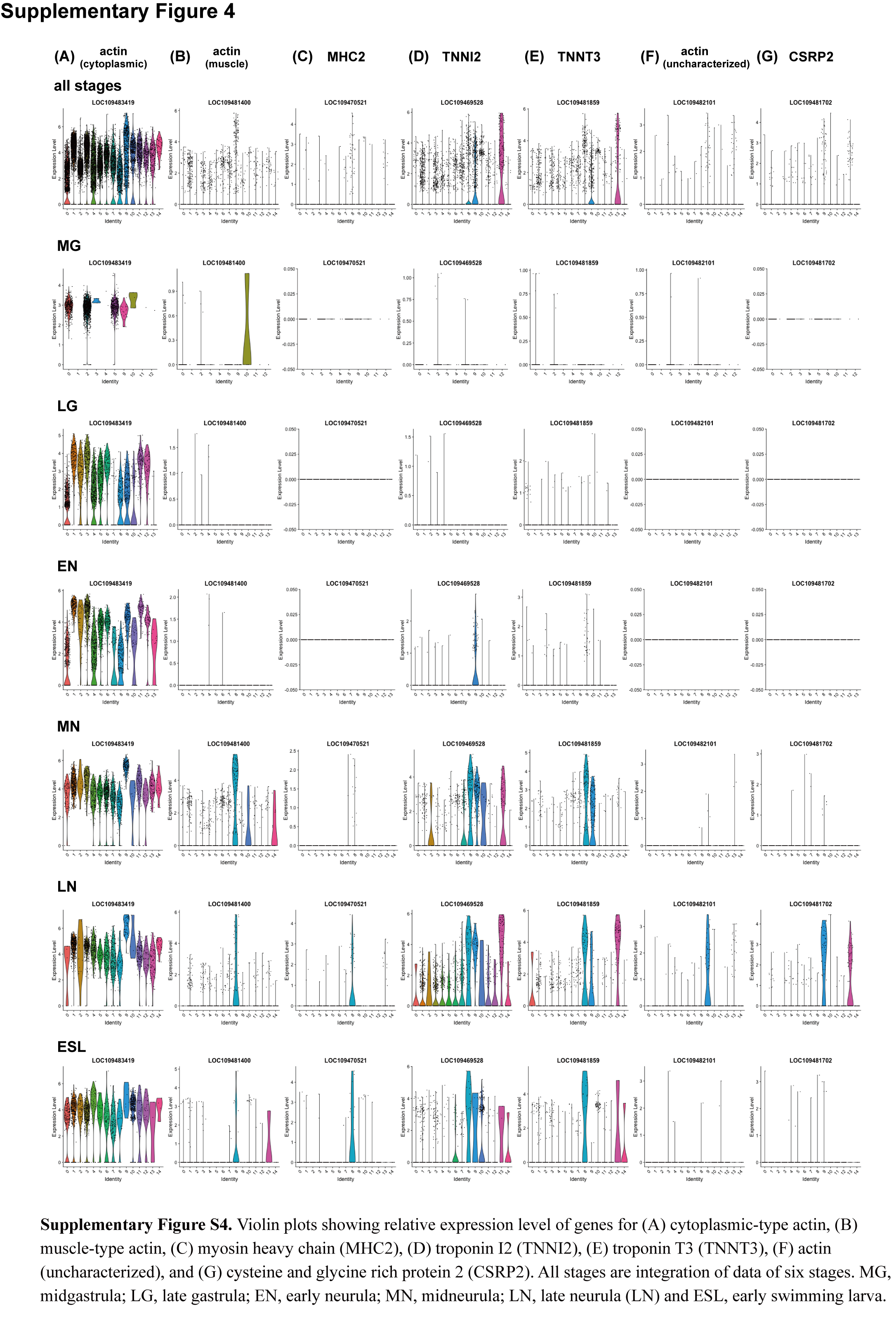

Supplement: Supplementary file 6 [file Image_3.TIF]

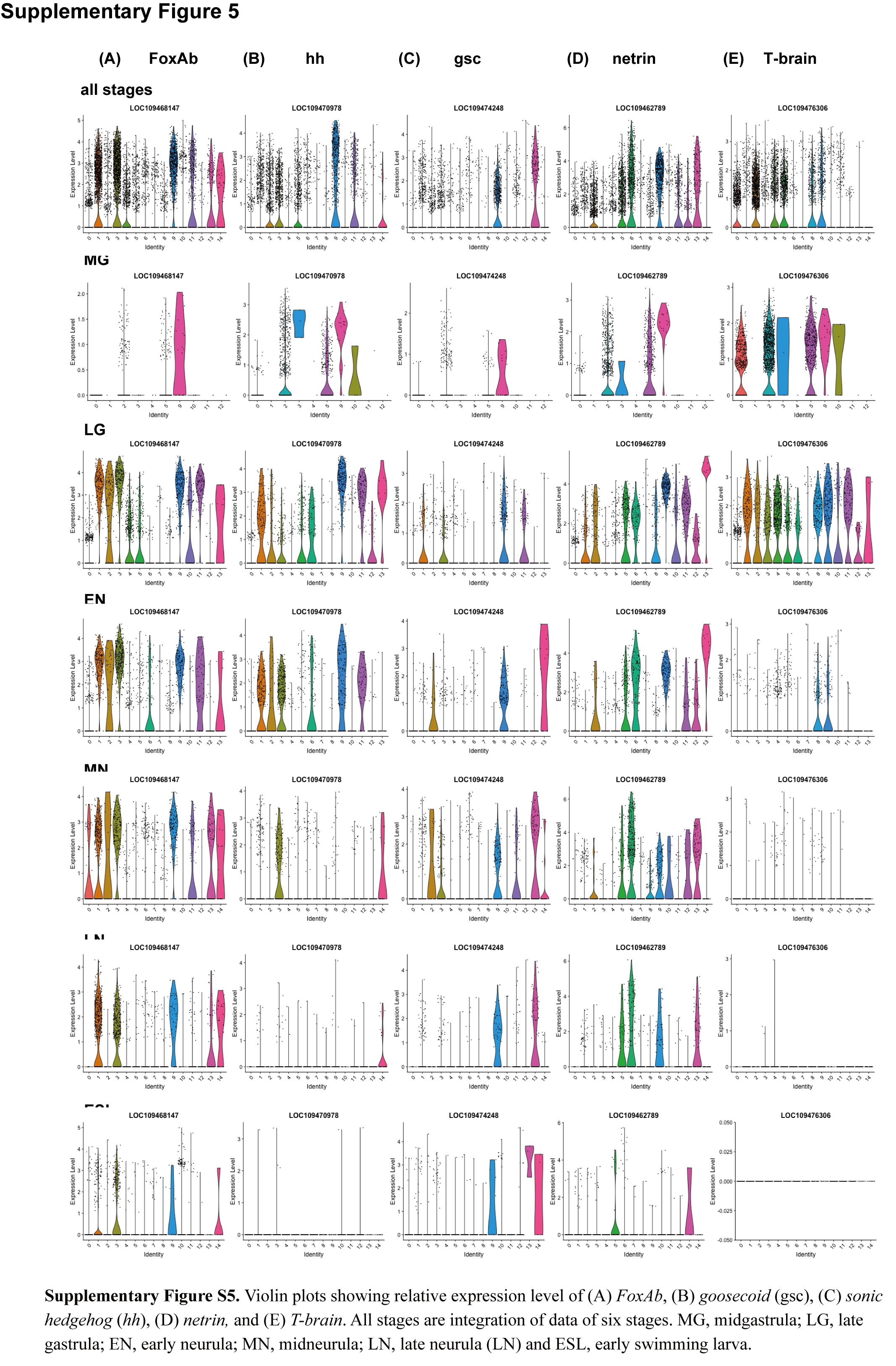

Supplement: Supplementary file 7 [file Image_4.TIF]

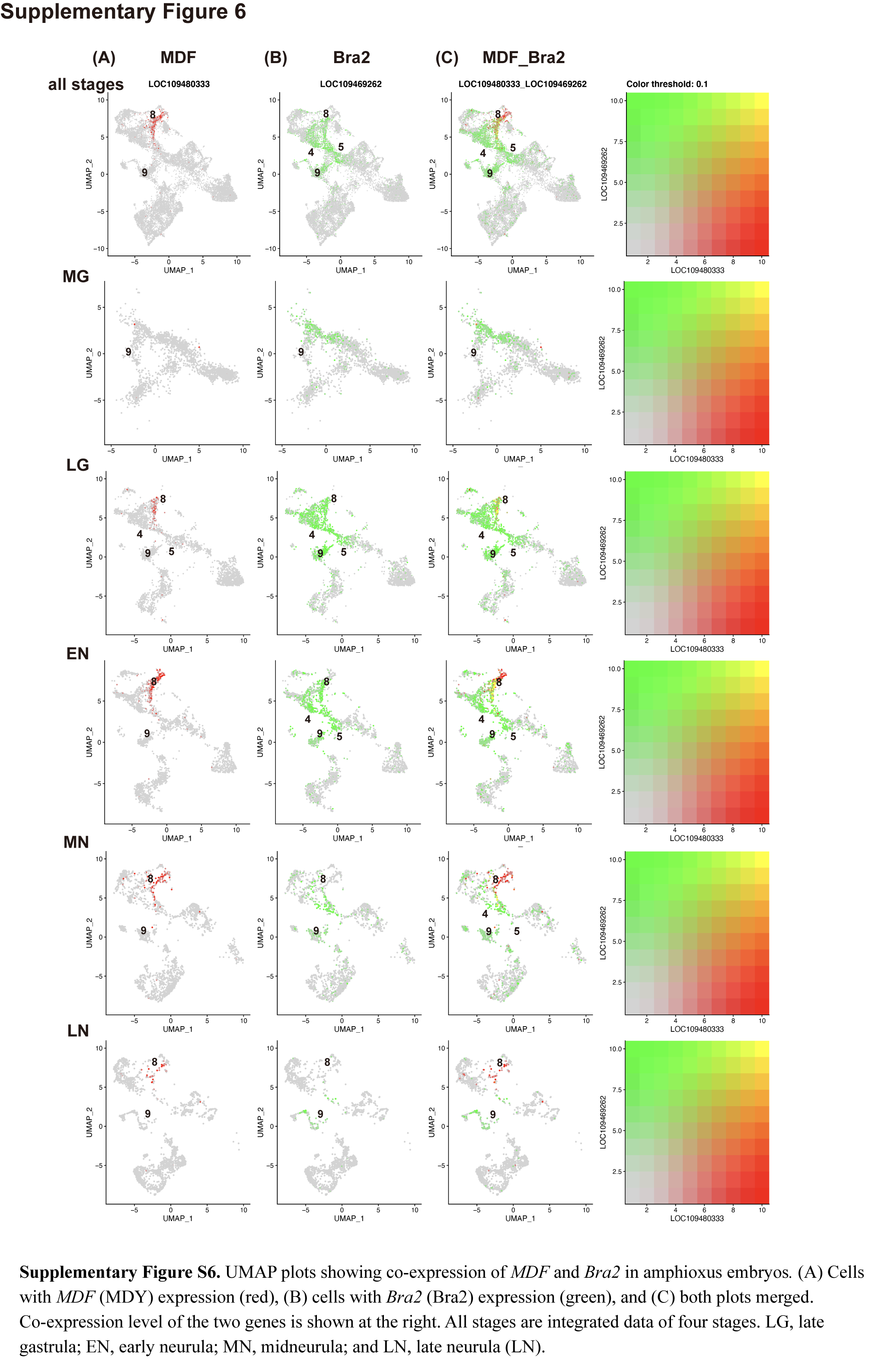

Supplement: Supplementary file 8 [file Image_5.TIF]

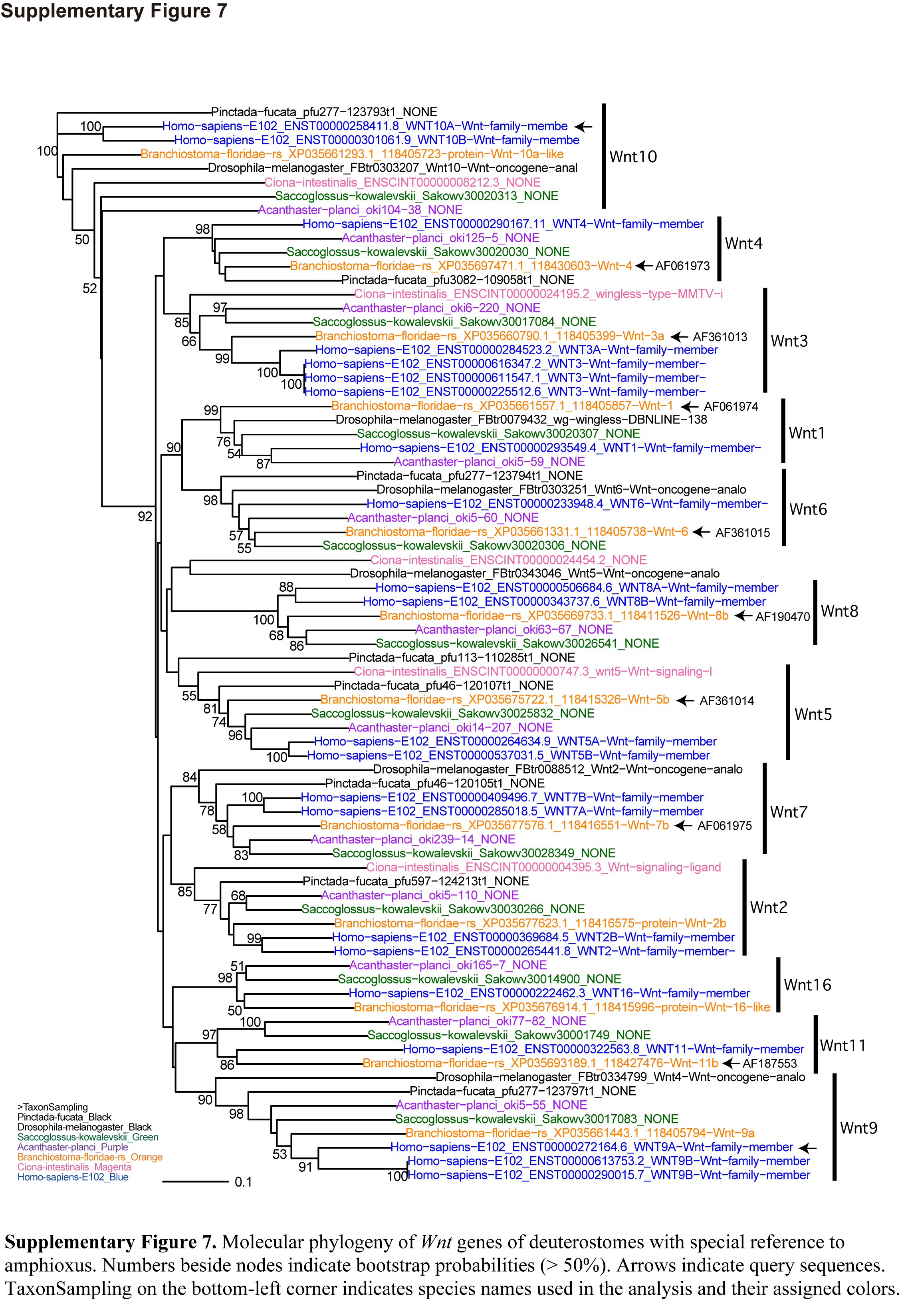

Supplement: Supplementary file 9 [file Image_6.TIF]

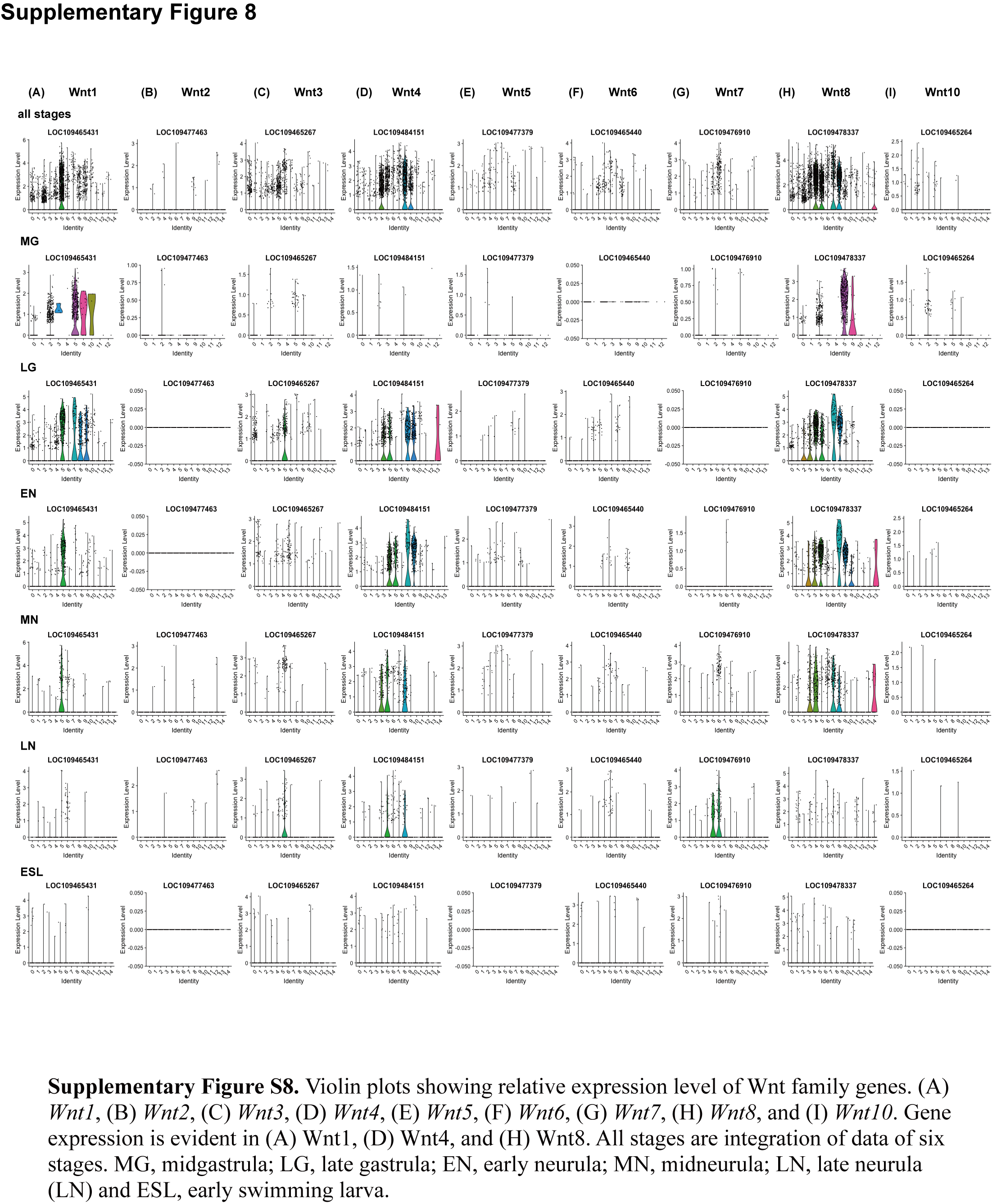

Supplement: Supplementary file 10 [file Image_7.TIF]

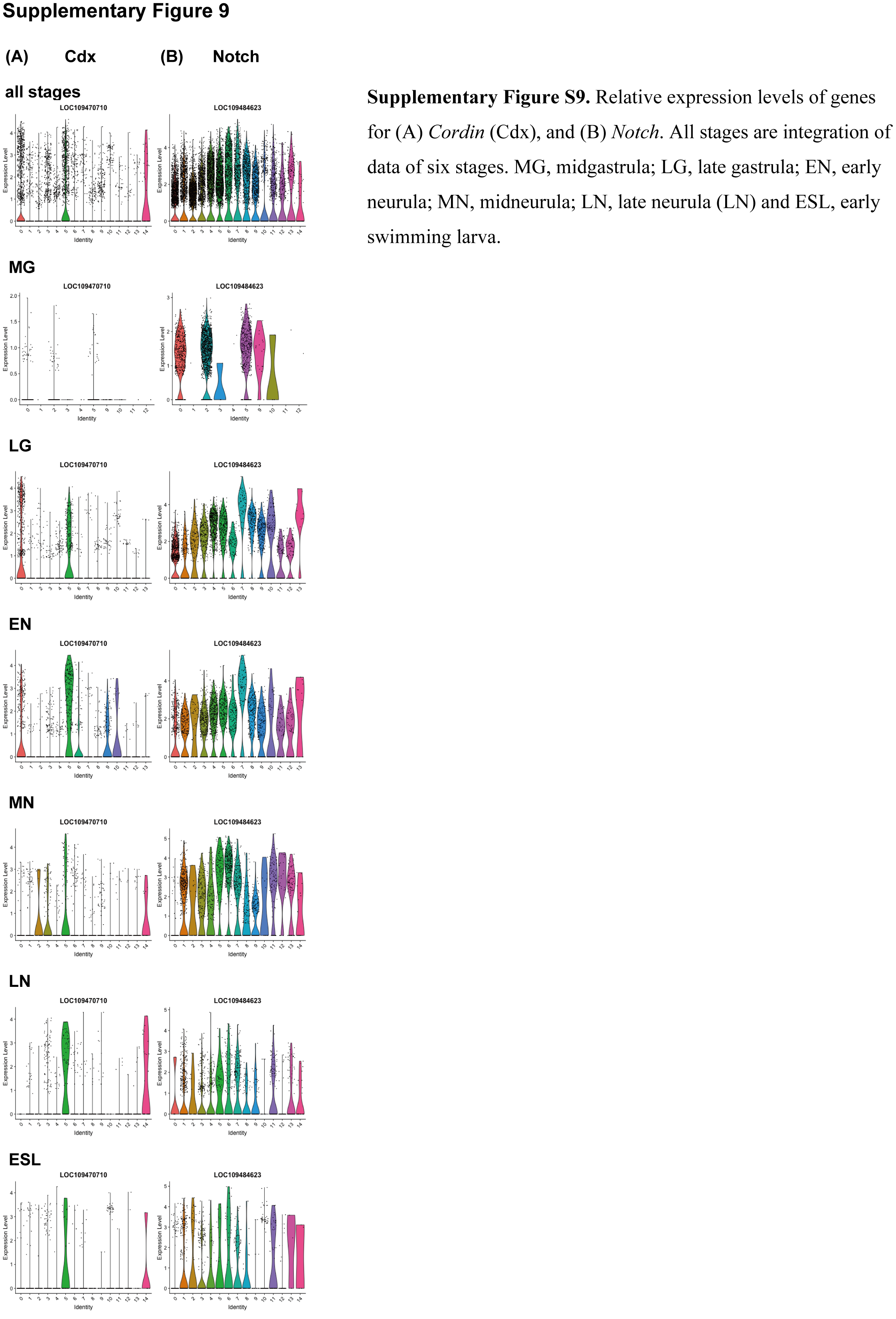

Supplement: Supplementary file 11 [file Image_8.TIF]

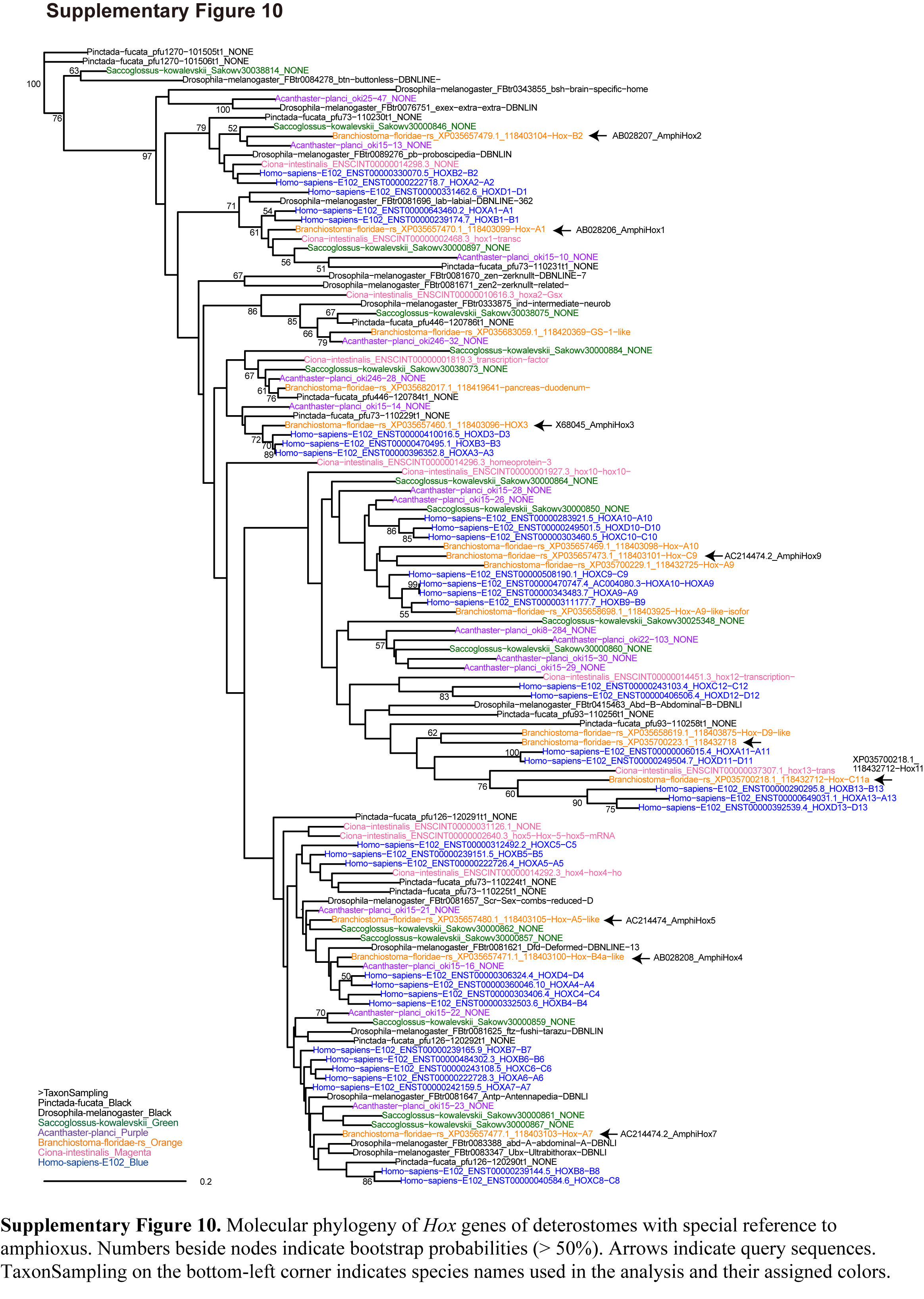

Supplement: Supplementary file 12 [file Image_9.TIF]

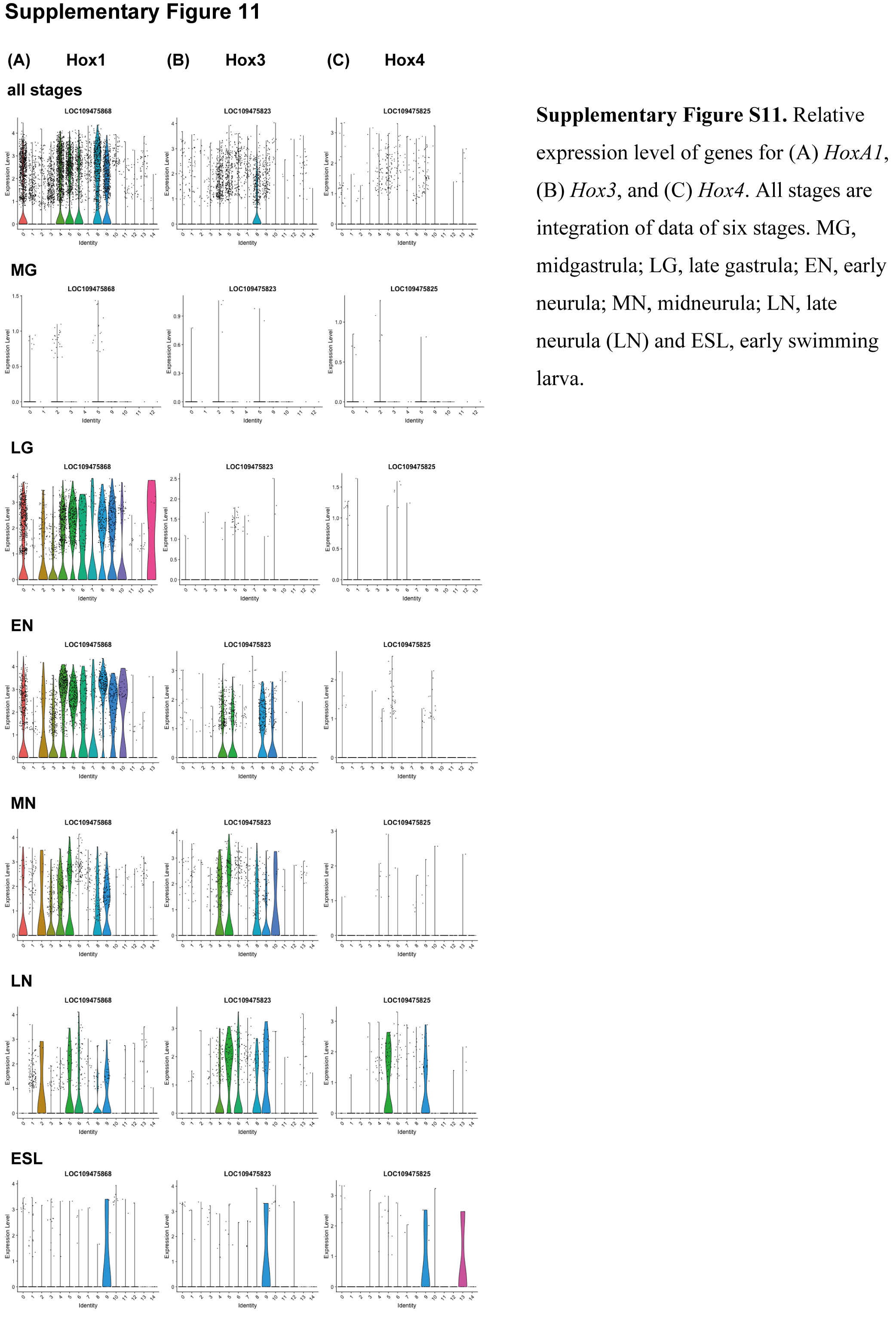

Supplement: Supplementary file 13 [file Image_10.TIF]
